# A Review of Nanocomposite-Modified Electrochemical Sensors for Water Quality Monitoring

**DOI:** 10.3390/s21124131

**Published:** 2021-06-16

**Authors:** Olfa Kanoun, Tamara Lazarević-Pašti, Igor Pašti, Salem Nasraoui, Malak Talbi, Amina Brahem, Anurag Adiraju, Evgeniya Sheremet, Raul D. Rodriguez, Mounir Ben Ali, Ammar Al-Hamry

**Affiliations:** 1 Professorship Measurement and Sensor Technology, Chemnitz University of Technology, 09111 Chemnitz, Germany; salem.nasraoui@etit.tu-chemnitz.de (S.N.); malak.talbi@etit.tu-chemnitz.de (M.T.); amina.brahem@etit.tu-chemnitz.de (A.B.); Adiraju.Anurag@etit.tu-chemnitz.de (A.A.); ammar.al-hamry@etit.tu-chemnitz.de (A.A.-H.); 2 Department of Physical Chemistry, “VINČA” Institute of Nuclear Sciences—National Institute of the Republic of Serbia, University of Belgrade, 11000 Belgrade, Serbia; tamara@vinca.rs; 3 Faculty of Physical Chemistry, University of Belgrade, 11000 Belgrade, Serbia; igor@ffh.bg.ac.rs; 4 NANOMISENE Lab, LR16CRMN01, Centre for Research on Microelectronics and Nanotechnology of Sousse, Technopole of Sousse B.P. 334, Sahloul, Sousse 4034, Tunisia; mounir.benali@issatso.rnu.tn; 5 Higher Institute of Applied Sciences and Technology of Sousse, University of Sousse, 4003 Tunisia of Sousse, GREENS-ISSAT, Cité Ettafala, Ibn Khaldoun, Sousse 4003, Tunisia; 6 Research School of Physics, Tomsk Polytechnic University, Tomsk 634050, Russia; esheremet@tpu.ru; 7 Research School of Chemical and Biomedical Technologies, Tomsk Polytechnic University, Tomsk 634050, Russia; rodriguez@tpu.ru

**Keywords:** electrochemical sensor, water contaminants, pesticides, inorganic compounds, emergent contaminants, in-situ applications, impedance spectroscopy, square wave voltammetry

## Abstract

Electrochemical sensors play a significant role in detecting chemical ions, molecules, and pathogens in water and other applications. These sensors are sensitive, portable, fast, inexpensive, and suitable for online and in-situ measurements compared to other methods. They can provide the detection for any compound that can undergo certain transformations within a potential window. It enables applications in multiple ion detection, mainly since these sensors are primarily non-specific. In this paper, we provide a survey of electrochemical sensors for the detection of water contaminants, i.e., pesticides, nitrate, nitrite, phosphorus, water hardeners, disinfectant, and other emergent contaminants (phenol, estrogen, gallic acid etc.). We focus on the influence of surface modification of the working electrodes by carbon nanomaterials, metallic nanostructures, imprinted polymers and evaluate the corresponding sensing performance. Especially for pesticides, which are challenging and need special care, we highlight biosensors, such as enzymatic sensors, immunobiosensor, aptasensors, and biomimetic sensors. We discuss the sensors’ overall performance, especially concerning real-sample performance and the capability for actual field application.

## 1. Introduction

The modern chemical industry is essential for providing sufficient goods and nourishment to the world’s population, but their excess may involve danger to humans, animals, and oceans, as highlighted by the European Environment Agency. More action is needed to tackle the mixtures of chemicals found in Europe’s waters [1]. According to [2], 38% of monitored lakes and 74% of the groundwater area achieved good chemical statistics, with pollutant concentrations not exceeding EU standards. Although 89 % of the sites has good qualitative status, 11% of waters are polluted, mainly due to contamination by nitrates from agricultural run-off, salt intrusion, and other hazardous chemicals from sources such as industrial sites, mining, or waste storage.

Rapid population growth, the uncontrolled application of chemical fertilizers, the heavy use of pesticides in agriculture and industry and domestic waste have resulted in an elevated level of pollutants in the environment. The continuously increasing rate of pollution affects the quality of drinking water, depleting aquatic systems and affecting the ecological cycle. The excessive use of fertilizers/pesticides in agriculture to balance the demand and use and discharge of plastics to water resources, hospitals industries effluents lead to water contamination and water-borne diseases. In this context, water quality monitoring is essential for detecting pollution and releasing toxic substances [3]. Hence, in 2020, the previous European Drinking Water Directive will be refreshed to incorporate new drinking-water safety criteria [4].

Electroanalytical chemistry has the potential to contribute significantly to the protection of the environment [5]. Recently, there has been an increasing interest in using electrochemical processes for water treatments [6]. Electrochemical sensors and detectors are suitable for on-site surveillance of critical contaminants. Electrochemical sensors are used in water quality monitoring of conductivity, dissolved oxygen, or pH. Their use has extended over the years to a broader range of environmental applications, notably detecting trace metals, carcinogens such as nitrogen and phosphorus compounds, and organic pollutants like phenols and pesticides [7].

An electrochemical sensor aims to deliver accurate and real-time information about a specific chemical composition in a particular environment and should be capable of responding continuously and reversibly without perturbing the sample. These devices consist of two key elements: a transducer covered with a chemical or biological recognition layer. For electrochemical sensors, the interaction between the target analyte and the sensitive recognition layer gives the analytical information. In the last years, new modified electrodes have been developed based on nanocomposites and highly selective biological or chemical detection layers. Several electrochemical sensors can be designed for environmental monitoring purposes depending on the chemical nature of the analyte to detect, the sample matrix and the requirement of sensitivity and selectivity. Lastly, the challenges of achieving repeatable and cost-effective methodologies and simple-to-use equipment for everyday analysis are pertinent. Such a wide range of possible applications proves the significance of electrochemical sensors in the evolution of environmental contamination detection.

Several reviews report on electrochemical sensing for water quality analysis [8,9]. Some of them focus on one type or a group of contaminants, such as the advances in nitrate monitoring in the environment and food products [10], the detection of phosphate -based on cobalt by potentiometric method and the progress of the development of electrochemical sensors for heavy metal ions [11,12,13]. Other reviews have combined several types of analytes, such as biomolecules (glucose, uric acid, dopamine, and ascorbic acid), pollutants (nitrobenzene, hydrazine, pesticides and nitrophenols), and heavy metal ions [13]. Also, some reviews focused on the materials used for sensing, such as carbon nanotubes [14], graphene [15], and polymers [16].

In this contribution, we provide an overview of the electrochemical detection of contaminants from the application of specific nanomaterials and technological points of view. This review focuses on electrochemical sensors used in field measurements or that have the potential for practical in-field water monitoring, including electrodes such as glassy carbon fabricated by microtechnology and printing technology. The review covers recent advances of stable, reproducible, and cost-effective electrochemical sensors towards water contaminant detection in the environmental and drinking water quality samples, focusing on in-situ, online and on-site measurements. The primary ecological contaminants are classified into various groups such as biological and chemical. These categories included a variety of subcategories that have a significant impact on the quality of the environment. In this review, the investigated targets are mainly pesticides, water hardness, disinfectants, nitrogen, phosphorus, and other emerging contaminants.

## 2. Water Contaminants

Pesticides, nitrate, nitrite, phosphorus, water disinfectant, phenolic compounds are the most notorious pollutants found in water. The quantity of these chemicals in surface water must be below the environmental quality standards defined by these directives to be considered in good chemical condition. The European Environment Agency European waters [17] measures to restrict the release of some of these compounds, such as NO_2_^−^, NO_3_^−^, gallic acid, 4-AP, Pb, and Ni, have been in place for many decades, but challenges remain in preventing the release of these contaminants in the environment and avoiding pollution from atmospheric compounds that are proven to be abundant, permanent, bioaccumulative, and poisonous continues to be a problem [17].

The European Commission has also released a watchlist of possible contaminants tracked in surface waters. An updated surface water watch list was adopted by the Commission [18]. Several drugs, hormones, and pesticides are currently on the list. The Drinking Water Directive strictly controls the amounts of pesticides in European drinking water. It contains the permitted quantities of pesticides, with a single pesticide limit of 0.1 µg/L and an actual number of pesticides quantified during the detection process of 0.5 µg/L. In addition to old mines and polluted industrial/waste sites resulting in contamination by As, Pb, Cu, and phenolic compounds, diffuse sources of emissions from agriculture (nitrates and pesticides) threaten groundwater. Table 1 summarizes some water contaminants and the corresponding guideline limit values.

Water contamination by emerging contaminants, such as pharmaceuticals, hormones, agrochemicals, and metallic and carbon nanomaterials, is a global issue. Their harmful effects on the environment and human health have already been proven [22,23].

### 2.1. Pesticides

Pesticides are substances designed and applied to repel, kill, or regulate harmful or interfering pests during the manufacture, processing, transport or marketing of foodstuffs, agricultural commodities, animal feed, or products that may be administered to animals to control insects, bacteria or other pests [24]. Pesticides play, therefore, an essential role in agriculture, enabling a significant increase in yields thanks to the eradication and control of pests. With the growing population, the use of pesticides has increased tremendously worldwide, with 45% of the usage coming from Europe itself, 25% from the USA, and the rest from the remaining countries [7].

Most pesticides accumulate in food, water, and the environment rather than directly reaching the target species. This unintended accumulation presents a severe threat to human health [3,25]. The active compounds present in the pesticide formulations are harmful to human health as they can cause anxiety, depression, convulsions, severe neurological disorders and cancer [26,27]. In addition, it disturbs the balance in the environment since the concentration of these toxic compounds is increasing exponentially [6]. For these reasons, there is a continuous drive for monitoring and quantification pesticides in the environment.

Pesticides can be classified according to their chemical structure as organophosphates, neonicotinoids, pyrethroids, carbamates, organochlorines [28], substituted phenols and triazines [29]. They can also be classified as herbicides, fungicides, insecticides and bactericides according to their targeted use [30]. Organophosphates and carbamates are the two main groups of pesticides mostly in use nowadays. Their toxic effects are attributed to the enzyme acetylcholinesterase inhibition [31]. Acetylcholinesterase (AChE) is an essential enzyme present in synaptic clefts of the central nervous system. The primary role of this enzyme is to hydrolyze acetylcholine, which is responsible for the proper functioning of the nervous system. AChE inhibition leads to acetylcholine accumulation and, consequently, hyperstimulation of nicotinic and muscarinic receptors and disrupted neurotransmission. [32]. Pesticide preparations available on the market nowadays contain different organophosphates and carbamates. Organophosphate preparations include, e.g., dimethoate, chlorpyrifos, and malathion. Carbamate compounds (carbaryl, carbofuran, carbosulfan) are the most widely used pesticides due to their high insecticidal activity and relatively low persistence.

### 2.2. Nitrogen

The nitrification process of the nitrogen cycle, carried out by microorganisms, produces nitrate and nitrite, which are among the naturally occurring chemical sources of nitrogen in ecosystems. Nitrate and nitrite are essential components of synthetic fertilizers, which aid plant growth to provide food for humans and animals. Via the conversion of dietary nitrates to nitric oxide, dietary nitrates have several beneficial effects on the human body, such as increasing blood flow, lowering blood pressure, and preventing cardiovascular diseases [33]. In addition, nitrite and nitrate are added during the production of foods to preserve them [34].

When nitrates reach the food chain from groundwater, surface water, and other sources, they can negatively affect the human body. If infants consume too much nitrate via their drinking water, they may develop “blue baby” disease or methemoglobinemia, which is caused by the endogenous conversion of nitrate to nitrite [10,35].

Water analysis shows nitrate concentrations in ground water are relatively low. In contrast, in agriculture and farming areas, nitrate-nitrogen concentrations are at the higher end and can exceed the 179.06 μM limit for drinking water. In some cases, nitrate-nitrogen concentrations exceed this limit, e.g., in wastewater sewage and landfills. Nitrite can react and be converted to nitrosamines that are carcinogenic in food and the human digestive system [36]. Nitrate is less toxic and more stable, but it can be converted to nitrite by chemical reduction in water [37], so there is a great need for detection and also continuous monitoring of these species because of their toxicity. For the electrochemical methods, the suitable modification of the electrode surface can improve the sensor response signals of nitrate and nitrite and extend the dynamic range in analytical determinations. Different materials have been reported to improve the sensor characteristics including carbon nanomaterials such as graphene [38] and carbon nanotubes [39], and metallic nanoparticles [40].

### 2.3. Phosphorus

Phosphorus control is considered to be a significant public issue to date due to its substantial industrial, environmental, health, and economic impact. Phosphorus (P) is present in the environment in either an inorganic or organic state. This nutrient is a life-giving element for the growth of flora and fauna, and lake ecosystems [41]. However, excessive amounts of inorganic phosphate (I-PO_4_) are among the main causes of growth and propagation of harmful algae known as eutrophication [42] of surface waters. Most inorganic phosphate is used as fertilizer for crop production and is thus released into the environment. Overall phosphorus levels (P-PO_4_) between 0.17 μM and 0.53 μM are assumed to be the limit values for eutrophication of surface water supplies [43]. In addition, I-PO_4_ is widely used in food processing (meat, seafood, beverage, bakery, vegetables) for many purposes such as humidity retention, pH regulation, protein dispersion, etc. Either a surplus or a deficiency of I-PO_4_ can lead to severe diseases affecting human health. A lack of I-PO_4_ (P-PO_4_ < 444.08 μM in adult serum), termed hypophosphatemia, indicates malnutrition. An excess of phosphate can lead to hyperphosphatemia (P-PO_4_ > 798.63 μM in adult serum), considered a risk indicator for chronic cardiovascular and renal disease mortality [44]. Reliable and inexpensive detection technology is of great interest for continuous monitoring to address these environmental, economic and health issues regarding the treatment and control of phosphates [45].

### 2.4. Water Hardeners

Water hardness mainly refers to the amount of dissolved minerals, especially calcium (Ca^2^^+^) and magnesium (Mg^2+^) in water, two divalent cations yielding insoluble carbonates, while their bicarbonates are soluble. Besides calcium and magnesium, some other divalent cations may also contribute to the total water hardness, such as manganese (Mn^2+^), iron (II) or strontium (Sr^2+^). However, in the majority of cases, the level of these cations is significantly lower than that of magnesium and calcium, so, in practical terms, they are typically neglected [46]. For example, in the case of calcium, general guidelines for classification certify that calcium carbonate is classified more than 180 mg/L as very hard [47].

Water hardness affects water consumption by populations for personal hygiene, food preparation and laundry (with an impact on soap consumption), especially in regions rich in carbonate minerals and therefore “hard” water.

### 2.5. Water Disinfectant Byproducts

Disinfection of water is a standard procedure in municipal water to control waterborne pathogens [48]. It is an essential step for the purification of water and to prevent water-borne diseases. The commonly used disinfectants for water purification are ozone, chlorine, chloramines and chlorine oxide [49]. However, the unavoidable reaction of disinfectants with natural organic matter and anthropogenic pollutants produces numerous byproducts, also termed disinfectant by-products (DBPs). These byproducts are responsible for adverse health effects on humans, such as cancer, reproductive and developmental effects [49]. Nevertheless, with the ever-increasing demand for purified water, the use of disinfectants has increased manifold leading to as many as 700 variants of by-products in drinking water [50].

The three main DBPs of the most significant health concerns are trihalomethanes, haloacetic acids and nitrosamines [48]. Trihalomethanes are four DBPs, namely trichloromethane, bromodichloromethane, dibromochloromethane and tribromomethane [48]. Haloacetic acids are classified into nine different types and termed HAA9 based on their reaction with the pollutants [51]. The disinfection by chloramines leads to the production of nitrosamines, which have four different types [48,52]. All these compounds are carcinogenic, highly toxic and cause irreversible damage to human bodies [53].

Apart from the DBPs mentioned above, which are strictly monitored, there are several other unregulated and newly emerging DBPs like halonitromethanes, iodo-acids and iodo-THMs, halonitriles, haloamides, halofuranones and haloaldehydes, to name a few [49]. Table 1 provides the maximum concentration limit of disinfectant by-products in drinking water according to United States Environmental Protection Agency (U.S-EPA), World Health Organization (WHO) and European Union (EU) guidelines.

### 2.6. Emergent Contaminants

Recently discovered groups of uncontrolled pollutants in surface water and groundwater commonly include daily use substances and various industrial additives. These substances are referred to as emerging contaminants (ECs). That raises a global environmental concern for water quality, posing severe potential threats to human health, wildlife, aquaculture life and ecosystems [54]. Such pollutants are usually bioactive and bio-accumulative and may have extensive occurrence and persistence. Global production of these contaminants is estimated to have been increasing from 1 million to 500 million tons per year [55]. The term of ECs could be referred to three main categories [56]. Firstly, substances recently released into the environment like industrial additives such as bisphenol A [57,58]. Secondly, chemical agents that could already have existed in the environment for several years, and only recently, their significance has begun to attract public attention, which is the case with pharmaceuticals [59], personal care products, etc. The third category concerns compounds already known for more extended periods, but whose potential harmful effects on public health and the environment have only recently been identified, for example hormones [60], steroids, surfactants etc. ECs sources are divided between specific point sources and diffuse sources [61]. Primary sources are households, industrial, hospital effluents and urban runoff.

**Figure 1:**
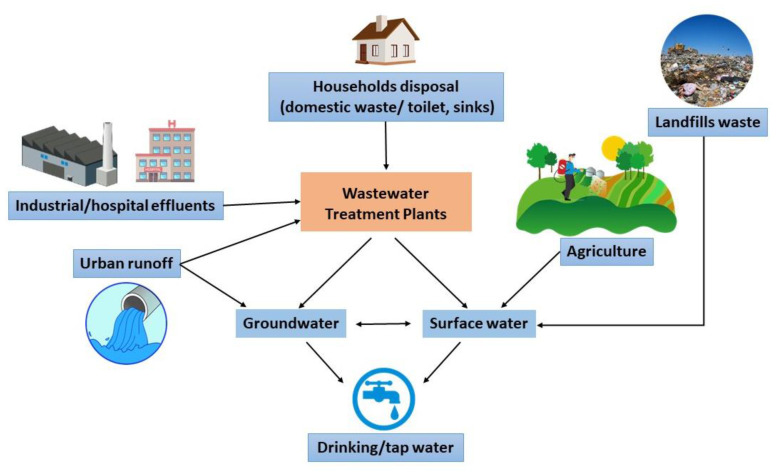
Primary sources of water contaminants in the environment.

Polyphenolic compounds (PCs) have received exceptional attention due to their harmful effect on the human body and the environment. Polyphenols are omnipresent secondary metabolites in foods [62]. They consist of phenolic hydroxyl group(s)-containing molecules, which are the basis of their antioxidant activity. In general, the reaction of this antioxidant action takes place with the loss of one electron to give a nontoxic, stable composition unable to propagate the response [63]. Antioxidants have many interactions in the food matrix, like preventing fat necrosis and reducing the harmful effects of nitrogen and active oxygen [63]. Several studies have been made to introduce sensitive and straightforward methods to evaluate antioxidant capacity (AOC) and quantify polyphenols in food. Figure 1 shows possible sources for emergent contaminants.

## 3. Electrochemical Sensor for Water Contaminants

### 3.1. Pesticide Sensors

Presently, high-performance liquid chromatography (HPLC) [64] and gas chromatography (GC) [28] coupled with mass spectroscopy (MS) are used to quantify various types of contaminants in the environment and food. These conventional measurement procedures are time-consuming, laborious, and typically require complex sample preparation and analyze the samples by comparing the obtained spectra with reference spectra [12,13]. Hence, there is a significant need for developing a fast, robust, low-cost, accurate and portable analytical system for the detection of pesticides.

The following text provides an overview of the electrochemical sensors for pesticide detection/quantification reported. We provide the analysis by dividing registered sensing platforms based on the receptor or active material at the working electrode.

**Figure 2:**
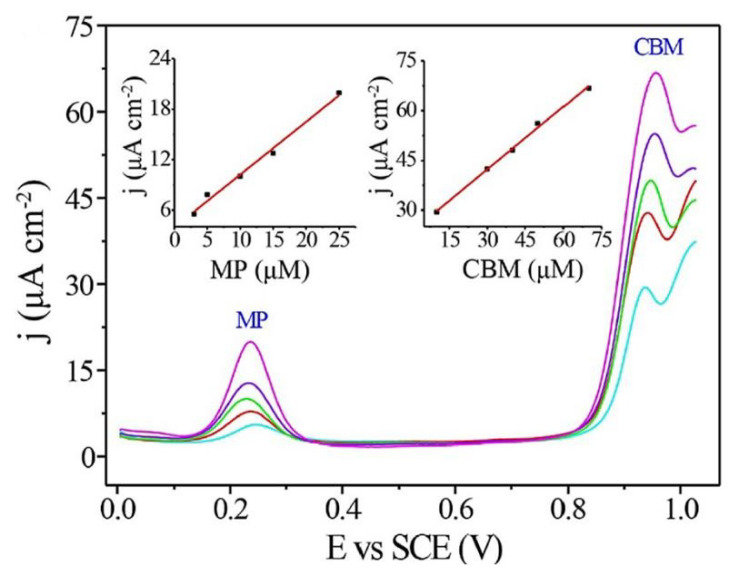
Simultaneous detection of the mixture of methyl parathion (MP) and carbendazim (CBM). The concentration ranges are (3–25) × 10^3^ μM for MP and (10–70) × 10^3^ μM for CBM. The insert profiles show the calibration curves between the peak current density and the target pesticide concentration. Reprinted from [70].

Bio-components warrant specific interactions of pesticides with sensors and have a relatively high selectivity. Electrochemical sensors rely on direct electrochemistry of pesticides and are connected with the electrocatalytic reaction of active electrode material(s) towards electrochemical transformations of analytes. For this reason, metallic materials are not something that is traditionally found in the field of pesticide detection and have appeared only recently. As a rule, the analyte must be electrochemically active on the electrode material so that the detection becomes possible. We also note that electrochemical sensors are not famous for their selectivity as any compound which can undergo an electrochemical transformation in the potential window where the analysis is done, can interfere with the analyte. Some of the first reports indicate differential pulse voltammetry to detect neonicotinoid pesticides (clothianidin, imidacloprid, thiamethoxam, nitenpyram and dinotefuran) using disposable screen-printed sensors with a sputtered bismuth film working electrode [65]. Limits of quantification (LOQ) were found to be in the range of 0.16 to 0.30 μM. In this work, significant efforts were made to reduce the matrix effect, and for this purpose, solid-phase extraction was employed. The use of silver amalgam electrodes was also demonstrated. Mercury meniscus0modified Ag amalgam electrode was used to detect tetrachlorvinphos with LODs amounting to 0.06 μM when differential pulse voltammetry is used and slightly better 0.04 μM when square wave voltammetry is employed [66]. Recently, an electrochemical microcell with Ag solid amalgam was employed for difenzoquat detection using differential pulse voltammetry. This approach enables the analysis of tens of microliter volumes with LOQ for difenzoquat below 0.45 μM L^−1^ [67]. Gold is irreplaceable in electrochemical laboratories for years, so it was also used for electrochemical detection of pesticides. Amperometric detection of the herbicide glyphosate with a LOD of 2 µM was demonstrated using gold electrodes [68]. According to US regulations this LOD is low enough to allow detection below the maximum permitted concentration in drinking water. Still, we note that glyphosate is not considered a very toxic compound for humans. More advanced approaches include the use of colloid gold for methyl parathion detection (LOD 10.5 × 10^−3^ μM) [69] and nanoporous gold for simultaneous detection of carbendazim (LOD 0.24 μM) and methyl parathion (LOD 0.02 μM) [70]. Simultaneous detection was possible due to a significant oxidation peak potential separation of the investigated pesticides (see Figure 2). 

Other examples of the use of gold-based electrodes, primarily nanosized gold on different supports, can be found, for example, gold nanoparticles/ethylenediamine-reduced graphene oxide for fenitrothion (LOD 6 × 10^−3^ μM [71]; Au nanoparticles supported by reduced graphene oxide for diuron detection (2.23 μM [72]; 3D graphene-Au composite for detection of 1-naphthyl methylcarbamate (LOD 0.0012 μM [73]; methyl parathion detection using pillar[5]arene@AuNPs@reduced graphene oxide hybrids (LOD 0.001 μM [74]; Au-Pd/reduced graphene oxide nanocomposite for parathion detection (LOD 0.008 µM [75].

There are examples of ultrasensitive detection of pesticides using Au-based materials. Dong et al. [76] reported a methyl parathion LOD of only 3.02 × 10^−5^ μM using a multi-walled carbon nanotubes-CeO_2_-Au nanocomposite in combination with stripping voltammetry.

According to the investigation, such a low LOD was due to the unique combination of high electrical conductivity and adsorption properties of multi-walled carbon nanotubes and high surface area and specific catalytic activity of Au and CeO_2_ nanoparticles. Appreciable performance was demonstrated also for gold nanoparticles/single-walled carbon nanotubes/glassy carbon electrode containing mono-6-thio-β-cyclodextrin (SH-β-CD) self-assembled monolayer [77]. With the use of square wave voltammetry LOD for methyl parathion was 10^−4^ μM with a linear response range from 2.0–80.0 × 10^−3^ μM. Interestingly, other frequently used pesticides like chlorpyrifos, 2,4-dichlorophenoxy acetic acid, methamidophos, triazophos, parathion showed very small effect on the analytical signal of methyl parathion.

Electrochemistry of pesticides using oxide-based electrodes is also a relatively new approach for pesticide detection. Methomyl detection using copper-oxide modified carbon paste electrode was demonstrated by Abbaci et al. [78] with a LOD of 0.02 μM. At the same time, CuO microspheres were employed for the detection of α-endosulfan using differential pulse voltammetry [79]. Very sensitive detection of diazinon, based on its direct reduction, enabling a LOD of 3 ×10^−3^ μM is possible using multi-walled carbon nanotubes covered by TiO_2_ nanoparticles [80]. In contrast, CuO-TiO_2_ hybrid nanocomposites were used to detect methyl-parathion with differential pulse voltammetry [81]. The LOD was, in this case, 1.21 ppb with basically no interference. Other oxide materials were also used, including NiO, to detect parathion with LOD of 0.024 µM [82]. CeO_2_-decorated reduced graphene oxide for determination of fenitrothion (LOD 3.0 × 10^−3^ μM [83]) and ZnO quantum dots for impedimetric detection of aldrin, tetradifon, glyphosate, and atrazine [84]. This platform was proposed for dual (tandem) measurements using the optical and electrochemical approaches. Some recorded low LODs were demonstrated using oxide materials for electrochemical detection of pesticides. The use of electrospun SnO_2_ for detection of atrazine enabled LOD of 0.9 zM [82]. Lately, several sensors for determination of methyl parathion were also reported, but with typical LODs for electrochemical detection in the submicro to the nanomolar range: 3D flower-like praseodymium molybdate-decorated reduced graphene oxide (LOD 1.8 × 10^−3^ μM), MnO_2_/PANI/rGO-A (LOD 7.4 × 10^−3^ μM) [85] and a monolayer of zirconium (IV) phosphonate on glassy carbon electrode (LOD 0.0045 μM) [86]. Also, the chitosan/magnetic Fe_3_O_4_ nanocomposite-modified glassy carbon electrode in combination with square wave voltammetry was used for bendiocarb determination, but the LOD was 2.09 μM with a LOQ of 6.97 μM [87]. Apparently, the selectivity was rather good, compensating for the relatively high LOD, and enabling bendiocarb detection in complex matrices.

#### 3.1.1. Sensors Based on Carbon Materials

Carbon materials are irreplaceable in electrochemical laboratories for many reasons. In principle, carbon materials are good electronic conductors, cheap, abundant, easy to work with, chemically inert and suitable for making composites, as can be already concluded from the previous discussion [88]. Generally, they are however rather bad electrocatalysts for water decomposition [89]. Many electrochemical reactions can be performed on carbon materials, while other materials would suffer from electrochemical transformations or cause electrolyte decomposition. However, their chemical inertness is a problem for electrochemistry. It is necessary to have an interaction between the electrode material and the analyte to perform electrocatalytic conversion and subsequent detection/quantification. Defects and functional groups in carbon materials, which are inherently present or intentionally introduced in the material, can be beneficial for the detection. Moreover, carbon with sp^2^ hybridized domains can interact with pesticides which have aromatic rings via π-π stacking interactions, enabling the necessary interaction with the analyte before the charge transfer step. Materials, which are traditionally used in electrochemical laboratories, are glassy carbon (GC), boron-doped diamond (BDD), and graphite materials from the carbon black (CB) family. Recently many carbon-based nanomaterials like carbon nanotubes (CNT) and graphene-based materials have been introduced. Some review papers on this topic are available in the literature [87]. While there are many cases where carbon materials are used as a support for different metallic or oxide nanostructures or scaffolds for biologically active compounds, several reports consider “only-carbon” electrodes for pesticide detection via their direct electrochemistry. In this case, similarly to the case when metallic and oxide materials are employed, typically more advanced electrochemical techniques are used to improve the detection capabilities of sensing platforms. So, instead of the standard cyclic voltammetry, differential pulse voltammetry or square wave voltammetry is employed. Typical detection limits and performances fall in the range of other electrochemical sensors for pesticides, while no record-breaking cases can be identified in this class of sensors.

Nevertheless, carbon-based materials are the most suitable for on-field use. These systems are generally very robust and rarely require special care due to the attractive properties of carbon materials. A selection of available literature reports and some of the characteristics of proposed sensing materials are summarized in Table 2.

#### 3.1.2. Sensors Using Molecularly Imprinted Polymers and Their Composites

**Figure 3:**
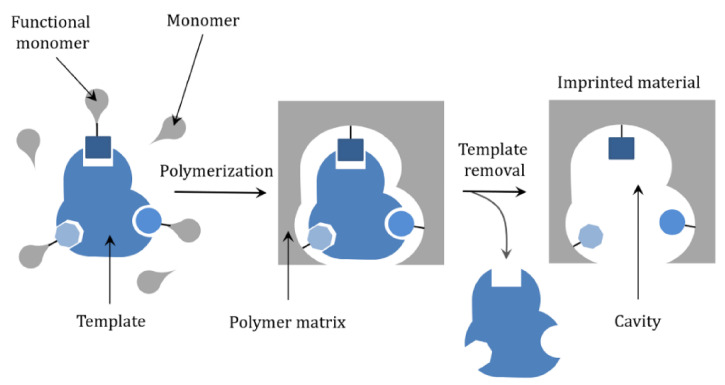
Preparation of molecularly imprinted materials.

Molecular imprinted polymer technology has attracted a lot of attention as it enables specific interactions of molecules with MIP structures. This section addresses MIP as a particular matrix for recognizing pesticides and does not consider their possible bio-like functions. The molecular imprinting technique relies on the formation of specially designed cavities within a cross-linked polymeric matrix (see Figure 3). Thus, particular guest-host interactions are operative and unique recognition of the analyte is possible. This means that selectivity increases while further electrochemical transformations of recognized analytes allow for high sensitivity and low detection limits. In electrochemistry, conductivity is essential so that many conductive polymers, like polypyrrole, are used in MIP technology. It is not surprising that conductive polymers are applied in this area, as they have been in use in electrochemistry labs for more than three decades. The knowledge accumulated on the electrochemistry of conductive polymers is used in this technology. The lessons learned from electrochemical polymerization studies are used to prepare specific MIP sensing platforms in the presence of an analyte template. While selectivity and sensitivity of sensors based on the MIPs approach that of immuno-based and aptasensors, we note that MIP technology is much more affordable and could resolve many of the problems associated with sensitivity and selectivity issues.

While specifically formed MIP structures are used to recognize target analytes, electrochemical methods used to generate the analytical signal are selected to improve sensitivity. One of the possible limitations of MIPs for electrochemical detection is that polymers are sometimes also electrochemically active. Their signal might be such a high that it masks the analyte’s signal, so this is something that must be considered when designing a sensing platform.

Other important issues are the possibility of irreversible electrochemical transformations of polymers and the pH sensitivity of polymers. Both issues might lead to irreversible losses of electroactivity, losses of conductivity, or conformational changes, which might disrupt recognition moieties of MIPs, compromising the sensor’s overall performance [108]. We summarize existing reports on MIPs for electrochemical pesticide detection in Table 3.

#### 3.1.3. Biosensors

(a) Enzymatic biosensors

Electrochemical biosensors are, historically, one of the first types of sensors employed for pesticide detection. Their action primarily relies on the inhibition of enzymatic components by the pesticide analyte and reduction of the electrochemical responses in the presence of analyzed pesticide. So, the indirect detection is so that the response in the presence of an analyte is compared to the response in the absence of an analyte. This is how an analytical curve is generated. Some of the first reports on this technique date back.35 years. Glass electrodes were modified with an enzymatic layer, enabling detection of carbaryl and azinphosethyl upon incubation of electrode with pesticide and enzyme-substrate [120]. Some of the first studies also used amperometric detection [121]. Several pesticides, including paraoxon, parathion, methyl paraoxon and methyl parathion, were coupled with acetylcholine and butyrylcholine esterases. Upon the contact of a pesticide with the enzyme, the activity was reduced. The detection principle involves observing the loss of enzyme activity in terms of choline production upon the exposure of inhibited enzymes to their substrates. Over the year, the research was directed either to demonstrate described detection principle for several pesticides or to improve detection limits by combining enzymatic components with different electrode materials, which show better electrocatalytic performance for detection of the products of enzymatic reactions. Using AChE detection, the detection limits for parathion and dichlorvos were 0.021 μM and 10^−4^ μM, respectively [122]. At the same time, the use of AChE immobilized on iron oxide (Fe_3_O_4_)/c-MWCNT/Au electrode enabled amperometric detection of malathion, chlorpyrifos, monocrotophos and endosulfan in the concentration ranges 0.1–40 × 10^−3^ μM, 0.1–50 × 10^−3^ μM, 1–50 × 10^−3^ μM, and 10–100 × 10^−3^ μM, respectively [123]. Other substrates for immobilization of AChE include nanocrystalline titanosilicate and ZSM-5 [124], iron oxide nanoparticles and poly(indole-5-carboxylic acid) for the detection of malathion and chlorpyrifos at nanomolar range [125] and gold nanoparticles obtained by electroless plating on three-dimensional graphene for detection of malathion and methyl parathion in water [126].

Another interesting example is the use of inhibitory action of chlorfenvinphos and malathion on lipase enzyme, whose substrate is *p*-nitrophenol acetate [127], and the product of the enzymatic reaction is *p*-nitrophenol. The product is detected using voltammetry, so the concentration of pesticides is linked to the reduction of *p*-nitrophenol production in the presence of pesticides. Detection limits were rather high, 84.45 μM and 253.03 10^3^ μM, for chlorfenvinphos and malathion, respectively, but this approach is rather new and amenable to further improvements. The same inhibition principle was used for fenitrothion using tyrosinase/poly(2-hydroxybenzamide)-modified graphite electrode [128], with catechol as the substrate. In this case, the detection limit was rather low, amounting to 4.70 × 10^−3^ μM. It is important to note that, traditionally, pesticide sensors based on enzyme inhibition (mostly AChE) relied on amperometric detection. However, recently, Malvano et al. [129] compared the performance of impedimetric affinity AChE-based biosensors to “standard” amperometric ones. While impedimetric detection gave ppb detection limits, the most interesting result is that the required analysis time is significantly reduced, from 20 min for amperometric to only 4 min for impedimetric detection. The principle of impedimetric detection relies on the impedance change upon the formation complex of pesticide with AChE, and it was demonstrated for water samples spiked with carbaryl and dichlorvos.

Another possibility is to employ enzymes whose substrates are organophosphates so that the products of the enzymatic reaction are detected using electrochemical methods. An old example by Mulchandani [130] demonstrated the use of organophosphate hydrolase (OPH) immobilized onto screen-printed carbon electrodes for ultra-sensitive detection of paraoxon and methyl parathion with detection limits of 0.09 and 0.07 μM, respectively. In this approach, organophosphate hydrolase produces *p*-nitrophenol during hydrolysis of OPs, which is easily detected by amperometry. The same principles were used for the development of biosensor arrays for micro- to nanomolar detection of OPs [131] and screen-printed, amperometric biosensor arrays for automated detection of several pesticides at 10^−3^ to 10 μM concentration range [131].

Further work showed that the use of cross-linked enzyme crystals of organophosphate hydrolase improves sensitivity and operational stability (particularly thermal) for detection of paraoxon compared to the use of enzyme in the non-crystalline form [132]. As in the case of cholinesterase-based biosensors, the catalytic action of the substrate for enzyme immobilization plays an essential role in the detection of p-nitrophenol as the product of OPs decomposition. A bionanocomposite sensing platform was recently demonstrated, consisting of chitosan-entrapped carbon nanotubes (CS-CNTs), ZrO_2_ nanoparticles, and OPH [133] was used for detection of paraoxon with a detection limit of 20 10^−3^ μM.

(b) Immunobiosensors

The application of immune-based components for pesticide detection is not as old an approach as the use of enzymatic biosensors but the first reports still date back 25 years. One of the first reports describes the development of a multichannel sensor for the detection of 2,4-dichlorophenoxyacetic acid with LOD of 0.0018 μM [134]. The same LOD was reported for the flow injection approach [135], while a LOD of 0.089 μM was reported for both 2,4-dichlorophenoxyacetic acid and 2,4,5-trichlorophenoxyacetic acid using potentiometric enzymatic immunoassay [136]. Other pesticides were also detected using immunobiosensors, including atrazine with biotinylated-Fab fragment K47 antibody [137] and glycine-doped polyaniline nanofilms on silicon with anti-atrazine antibodies [138]. LOD was of the order of μg per liter. Recently, parathion was also detected using electrochemical immunobiosensors. Impedimetric measurements by graphene-modified screen-printed immunosensor led to one LOD of 9.31 10^−8^ μM [139]. In contrast, the use of graphene quantum dots led to further improvements of LOD and its reduction to 8.23 10^−8^ μM [140]. However, the most astonishing is LOD of atrazine using electrospun Mn_2_O_3_ nanofibers modified with atrazine antibodies. The LOD amounted to only 3.93 × 10^−15^ μM with impedimetric detection [141].

Microorganisms are also used as platforms for electrochemical pesticide detection. This strategy is also relatively new. The general principle relies on the toxic effects of pesticides on living cells and measuring the products of their metabolism. These systems seem particularly suitable for monitoring overall contamination as the complexity of living cells, and their responses to particular compounds seem like a big challenge for obtaining high selectivity. Amperometric detection was combined with a platform formed of *Saccharomyces cerevisiae* cells on a combination of polyvinyl alcohol hydrogel and sodium alginate as a matrix at the electrode surface for measuring toxicity of acephate, ametryn and thiram [142]. Also, *Escherichia coli*, *Shewanella oneidensis* and *Methylosinus trichosporium OB3b* immobilized on screen-printed gold electrode surface were used for monitoring water pollution. At the same time, the possibility to integrate sensors in sensor arrays was outlined in Abu-Ali et al. [143]. Recently, the use of an artificial neural network approach for identifying pollutants using bacteria immobilized on gold screen printed electrodes was demonstrated, including detection of pesticides (atrazine), heavy metal ions, and petrochemicals [144].

(c) Aptasensors

**Figure 4:**
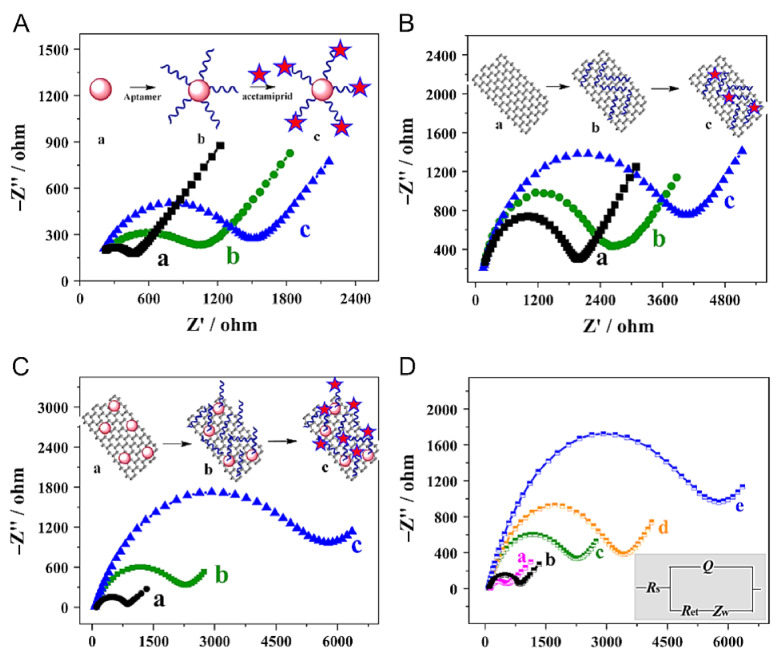
Comparison of the EIS responses for the GCE modified with different nanomaterials before and after aptamer immobilization and acetamiprid detection: (**A**) AuNPs/GCE, (**B**) MWCNT-rGONR/GCE, (**C**) Au/MWCNT-rGONR/GCE; (**D**) EIS responses of bare GCE (a), Au/MWCNT-rGONR/GCE (b), aptamer/Au/MWCNT-rGONR/GCE (c), MCH/aptamer/Au/MWCNT-rGONR/GCE (d) in 0.1 M PBS (pH 7.0) containing 5 mM [Fe(CN)_6_]^3−/4−^ and 0.1 M KNO_3_, and EIS responses of MCH/aptamer/Au/MWCNT-rGONR/GCE (e) after 100 nM acetamiprid captured on the modified electrode in 0.1 M PBS (pH 7.0) containing 5 mM [Fe(CN)_6_]^3−/4−^ and 0.1 M KNO_3_. Reprinted from [145].

Aptamer technology is relatively new, so the first reports date back to only four years ago. However, superior performance is demonstrated. An impedimetric aptasensor using gold nanoparticles decorated multiwalled carbon nanotube-reduced graphene oxide nanoribbon as a platform for immobilizing aptamer (see Figure 4) demonstrated an ultra-low LOD for acetamiprid of only 1.7 10^−8^ μM [145]. Using Pt nanowires as the platform for aptamers, in combination with impedimetric measurements, the same pesticide could be detected with a LOD of 10^−6^ μM, while atrazine was detected with a LOD of 10^−5^ μM [146]. These detection limits are much lower compared to the ones obtained using enzyme-based biosensors. Reusable screen-printed carbon electrodes were recently developed to detect organophosphate pesticides based on graphene oxide-ferroferric oxide combined with Aptamer. Polydimethylsiloxane was used to avoid the adsorption of molecules on the surface of the working electrode. It was concluded that the aptasensor could be used twice without signal loss and effect of interferences [147].

(d) Biomimetic sensors

This is one of the newest sensor technologies, and only a couple of reports can be found. An astonishing performance of electrochemical biomimetic sensor based on functionalized gold nanoparticles with an oxime group and nitrogen-doped graphene composites which detects dimethoate with LOD of only 8.7 10^−13^M, was demonstrated by Zhang et al. [145], indicating performance close to that of aptasensors. Another example is the use of functionalized a polyacrylamide, polyhydroxamicalkanoate, which mimics AChE. Using this platform for amperometric detection of pesticides paraoxon-ethyl (LOD 0.36 μM), fenitrothion (LOD 0.61 μM), and chlorpyrifos (LOD 0.83 μM) were effectively detected [148]. A bifunctional nanoenzyme, cerium oxide, mimicking organophosphate hydrolase behavior, was used to decompose methyl parathion to *p*-nitrophenol, and the same nanoenzyme coated on the surface. The obtained LOD for methyl parathion was 0.06 µM. As one can see, the LODs are close to those of enzymatic biosensors, but, importantly, species that usually interfere with enzymatic sensors do not affect the performance of biomimetic systems.

**Figure 5:**
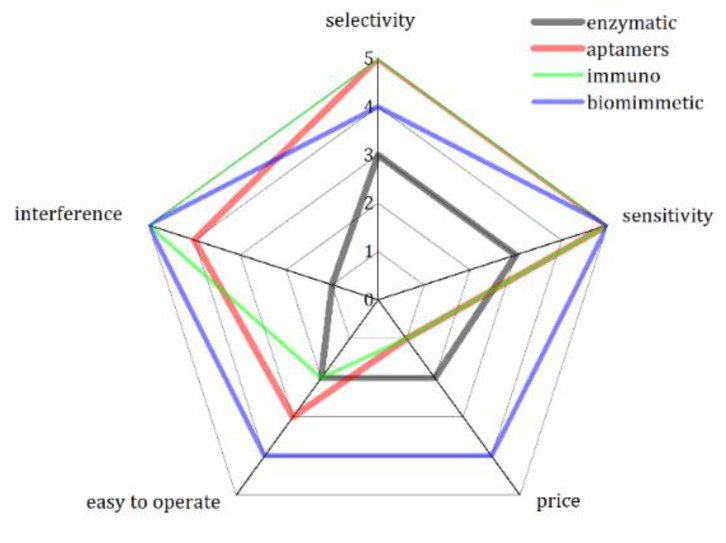
Spider diagram for the overall performance of different electrochemical biosensors for pesticide detection (1—bad, 5—very good). Remark: We do not consider sensors based on living cells.

As a conclusion on electrochemical biosensors for pesticide detection, we note that special molecules require special care, so it is hard to imagine a non-properly educated person working with bio-based sensors in the field. New technologies that appeared some 5 years ago in this field such as aptamers and biomimetic systems offer more robust solutions. These also hold the record, as we see, in terms of sensitivity. While any compound, which inhibits the enzymes, could interfere with enzymatic biosensors relying on enzyme inhibition, this problem is resolved using specific interactions with immuno-based components. One of the best examples is the detection of atrazine using immuno-based impedimetric detection, realizing a low LOD [141]. However, this solution comes with a great price, and these sensors cannot be considered very affordable. An overview of the performance and maturity of available technologies related to bio-based sensors for pesticide detection is given in Figure 5.

#### 3.1.4. Sensors Based on Other Materials

Besides the main classes of materials and recognition components described above, there are examples of some atypical materials or atypical combinations of materials applied for building electrochemical sensing platforms for pesticides. For example, metal-organic frameworks (MOFs) have received a lot of attention in many different fields, including analytics, so it is not surprising that MOFs were also investigated for pesticide quantification. Karimian et al. [149] applied TiO_2_-functionalized graphene oxide to modify MOF UiO-66 for the simultaneous determination of paraoxon and chlorpyrifos. Appreciable performances were observed, with LODs of 0.2 10^−3^ μM (paraoxon) and 1.0 10^−3^ μM (chlorpyrifos), and the corresponding linear ranges of 1.0–100.0 10^−3^ μM and 5.0–300.0 10^−3^ μM, respectively, with the use of square wave voltammetry.

MOFs might be new hot materials in this field as they combine precise and open pore structure and a possibility for functionalization in order to improve catalytic (charge transfer) properties and electrical conductivity, which must not be disregarded when talking about electrochemical sensors. Some other examples of rarely investigated classes of materials are well known in other fields of electrochemistry. One example builds metallic phosphosulfides, resulting from electrocatalysis of hydrogen production. Paraoxon ethyl was detected and quantified using graphene-based NiFe bimetallic phosphosulfide nanocomposite with a rather low LOD of 3.7 10^−3^ μM [150]. The synergistic effect of π-π stacking interactions has been emphasized between the aromatic moiety of paraoxon ethyl and graphene and the strong catalytic effect of NiFe phosphosulfide. Another example is a carbon black-cobalt phthalocyanine (CoPc) nanocomposite. CoPc and other transition metal phthalocyanine are intensively investigated for oxygen reduction reaction as an alternative to Pt, while Cinti et al. [151] demonstrated low LOD of paraoxon (18 nM) using the mentioned composite at screen-printed electrodes. Even lower LODs were found for methyl parathion (3.1 10^−7^ μM), diazinon (6.7 10^−8^ μM) and chlorpyrifos (3.3 10^−8^ μM) in water samples using graphene oxide decorated with monodisperse boron nitride quantum dots [152]. Further examples can be found in [153,154,155].

### 3.2. Water Hardness Ions

Water hardness describes the total concentration of alkaline earth ions in water [156]. In real water samples the concentrations of calcium and magnesium are usually much higher than other ions, so commonly, hardness often refers to the sum of the calcium and magnesium ion concentrations. The detection of other ions like magnesium (Mg^2+^) is more challenging since they are present in small amounts.

Titration is nowadays the standard method for the determination of water hardness. Fluorimetry and capillary electrophoresis are also used for the determination of water hardness [155]. However, these methods have problems with complex operation procedures and expensive instruments. Ion-selective electrodes (ISEs) can provide inexpensive, simple and continuous measurements of water hardness [157,158].

Recently, screen-printed ISEs have attracted significant attention to detect several ions such as Pb^2+^, K^+^ and Ca^2+^ [159]. Due to their high cost-effectiveness and simple manufacturing processes, screen-printed electrodes (SPEs) offer unique advantages for real-time monitoring for industrial, clinical, and environmental analysis. There are many types of SPEs, one of them is potentiometric.

Electro-active materials are therefore sought and implemented as solid contacts like graphene and carbon nanotubes during the manufacturing of all-solid-state ISEs, to enhance the stability of electrode potential. An example is an integrated Ca^2+^ potentiometric strip for which electrochemical and physical techniques previously treat the conductive carbon ink-based ceramic substrate. Potentiometric and electrochemical performances strip have been investigated in seawater [160]. Potentiometric Ca^2+^ determination using screen-printed ASS sensors can be an advantageous alternative with interesting accuracy and precision compared to conventional chromatography and photometric techniques. Herein, an example of electrochemical performance of planar miniaturized all-solid-state (ASS) screen-printed potentiometric sensor to detect Ca^2+^ ions in real water samples [161]. In the same context, another potentiometric solid-contact ion-selective electrode (SC-ISEs) has been developed for the detection of Ca^2+^ using the nanocomposite of ordered bimetallic AuCu NPs coupled with multi-walled carbon nanotubes (oAuCuNPs-MWCNTs) as a transducer [162]. This investigation demonstrates a general and facile approach to develop robust and durable solid-contact ion-selective electrodes based on nanocomposite as transducing layers to meet the requirements for the clinical and environmental “in-situ” potentiometric detection.

The electrochemical methods for magnesium (Mg^2+^) detection are still under investigation and need more research effort since very low concentrations need to be detected.

Most electrochemical sensors for calcium and magnesium are generally ISEs. Also some research succeeded in investigating both analytes simultaneously, as mentioned in Table 4.

### 3.3. Nitrogen Sensors

The transducer functionalization and modification affect the sensor surface response by improving the output signal, reducing the overpotential of nitrite, enlarging the linear range, and enhancing the sensor characteristics such as the limit of detection and sensitivity and selectivity. Carbon nanomaterials, metal nanoparticles, and conducting polymers have promising physical and chemical characteristics improving the working electrode surface conductivity and the electrocatalytic activity toward the target. Besides, integrating two nanomaterials or more to obtain a hybridized composite can be an alternative sensitive layer for the sensor modification. As a result, the catalytic performance of the composite material is further enhanced because of the synergistic effect. Table 5 shows selected developments in the field of sensitive materials for the functionalization of working electrodes toward the detection of nitrate and nitrite published during the last five years.

#### 3.3.1. Carbon-Based Materials

**Figure 6:**
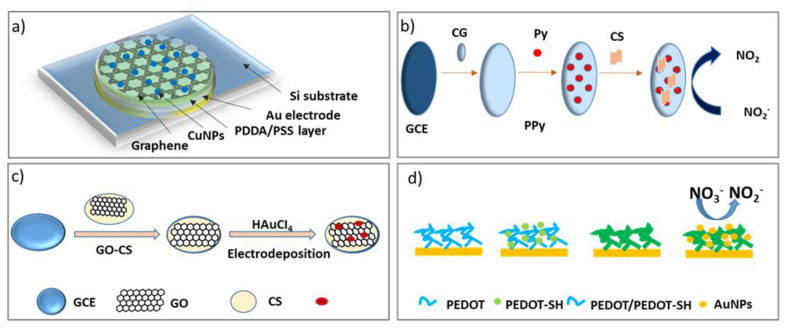
Redrawing of (**a**) self-assembled graphene and copper nanoparticles composite sensor structure following reference [190], (**b**) fabrication process of CG/PPy/CS/GCE for sensitive detection of NO_2_^−^ following reference [201]. (**c**) the construction process of graphene oxide (GO)-chitosan (CS)-Au nanoparticles (AuNPs)/glassy carbon electrode (GCE) [177] and (**d**) Growth process of PEDOT/PEDOT-SH/Au on electrode surface [210].

Graphene is a crucial promising two-dimensional carbon nanomaterial presenting several advantages; excellent conductivity and catalytic activity [200]. Metal nanoparticles [170,201], can be functionalized together with graphene and used as a sensitive layer for the electrochemical sensor for nitrite and nitrate detection. Wang et al. [190] have developed a screen-printed electrode to detect nitrate in lake water (see Figure 6a). Combining graphene and Cu NPs can improve the sensor response, which presents a limit of detection of 7.89 μM. Xio et al. [201] have developed graphene and chitosan (CS) nanocomposite (see Figure 6b). due to the better conductivity and absorbability of the prepared graphene-based composite. The developed sensor has good reproducibility, a limit of detection of 0.02 μM in the linear concentration range between 0.2–1000 μM was obtained. Since graphene has limited performance on its own, it is often combined with metamaterials in composite nanomaterials [202].

Carbon nanotubes (CNTs) also is an essential carbon-based material. Due to its superior characteristics, electrical conductivity, empty cylindrical structure, large specific surface area and good electrocatalytic activity, etc. it is considered that the blend of CNTs and metal nanomaterials, for example, the decoration of CNTs by metal nanoparticles, additionally enhances the electrochemical response in the presence of CNTs by accelerating the electron transfer on the electrode surface. Thirumalraj et al. [203] reported on an amperometric electrochemical nitrite sensor using functionalized MWCNT decorated by palladium nanoparticles (PdNPs). The PdNPs were fixed into the carbon nanotubes modified glassy carbon electrode surface by the electrodeposition method. A f-MWCNT/PdNPs composite-based sensor was applied to detect nitrite in water samples. It showed satisfactory results, a detection limit of 22 10^−3^ μM and a wide linear range of concentration from 0.05−2887.6 µM. It also showed long-term stability, good selectivity toward interferences, high reproducibility, and applicability in different real water samples with high accuracy. Another sensor has been reported by Bagheri et al. [172] for the simultaneous monitoring of nitrite and nitrate in the tap and mineral waters, sausages, salami, and cheese samples. The sensor is based on copper nanoparticles decorated multiwalled carbon nanotubes–reduced graphene oxide nanocomposite modified glassy carbon electrode (Cu/MWCNT/RGO/GCE). Under optimized conditions the Cu/MWCNT/RGO/GCE shows a limit of detection of 30 10^−3^ μM and 20 10^−3^ μM for nitrite and nitrate ions, respectively, in the range from 0.1−75 µM.

#### 3.3.2. Metal Nanomaterial

Metal nanoparticles, such as gold, silver, platinum, palladium, copper, bimetallic and so on, were widely used to fabricate nitrite electrochemical sensors with increased sensitivity according to their excellent catalytic activity and conductivity unique structure and high specific surface area.

Fajerwerg et al. [204] electroreduced nitrate at the surface of a silver nanoparticle (AgNPs)-electrodeposited gold electrode. Chronoamperometry was used to generate the AgNPs. The charge (Q) used here for the electrodeposition of AgNPs is lower than the charge used for the fabrication of the silver monolayer. The proposed sensor AgNPs/gold electrode shows a low limit of detection of 10 µM because of the interaction of two chemical reactions and electron transfer at the linear concentration range from 10–10^3^ µM.

AgNPs were used as sensitive material for detecting nitrate in the investigation of Ghanbari. Silver nanoparticles dispersed in polypyrrole matrixes coated on glassy carbon electrode as a nitrate sensor [205]. The AgNPs were easily synthesized by electrodeposition onto the surface of polypyrrole (PPy) matrixes modified glassy carbon electrode (GCE). The sensor presented an excellent electrocatalytic activity for nitrate reduction, a limit of detection for nitrate of 2.0 μM and a range of concentration from 2 to 100 μM. Also, the sensor showed good real sample results in cheese sausage samples extracted in water and mineral water samples with excellent sensitivity, selectivity, and stability.

Xi et al. [206] reported a nitrite electrochemical sensor by depositing nanocomposites of palladium and platinum (Pd-Pt) on the surface of porous gallium nitride (PGaN). The Pd-Pt/PGaN sensor combination presents an excellent electrocatalytic activity for nitrite monitoring, with a detection limit of 0.95 µM at two different ranges of concentration from 1 to 300 µM and from 300 to 3000 µM.

Mo et al. [177] developed an electrochemical sensor based on Au nanoparticle electrodeposited onto a graphene-chitosan-modified glassy carbon electrode (see Figure 6c). The prepared sensor presents 0.3 µM as detection limit at the range from 0.9 µM−18.9 µM. Due to the high sensitivity of silver nanoparticles toward nitrate ions, Bonyani et al. have proposed silver nanoparticles/polymethacrylic acid nanocomposite (AgNPs/PMA) modified screen-printed electrode AgNPs/PMA/SPCEs [207]. The AgNPs/PMA/SPCEs combination based on silver dendritic structures showed a sensitivity of 130 µA mM^−1^ cm^−2^ at the range from 0−20 × 10^3^ μM.

It can be concluded that the functionalization of the working electrode with nanostructured metallic materials plays an essential role in enhancing the electrochemical sensing performances and improving the sensor characteristics.

#### 3.3.3. Conducting Polymers

Conducting polymers, such as polyacetylene, polypyrrole, polythiophene, polyaniline and their derivatives have received considerable attention for their unique metal and semiconductor-like properties compared with traditional polymers [208]. Due to their favorable mechanical, optical, electrical, and electrochemical characteristics, CPs have been widely used to fabricate sensors, biomedical and microfluidic devices, etc. Polyaniline (PANI) has been presented as a sensitive material toward nitrite and nitrate ions because of its characteristics, including conductivity, environmental stability, etc. Diarisso et al. [209] synthesized a nitrite sensor using PANI. The aniline monomer was electropolymerized after the electrodeposition of 4-aminobenzenesulfonic diazonium salt (ABS) on the surface of glassy carbon electrode PANI/ABS/GCE. Furthermore, the PANI/ABS/GCE showed a 23.04 µA µM^−1^ cm^−2^ and a detection limit of 0.48 μM at the range of nitrite concentration from 0.5 and 35 μM, therefore good reproducibility. Yi et al. [210] functionalized a glassy carbon electrode using gold nanoparticles multilayered film of poly(3,4-ethylenedioxythiophene) (see Figure 6d). The film at the electrode surface possesses a large electroactive surface area and excellent sensitivity for nitrite detection. The proposed electrode combination PEDOT-SH/Au/GCE showed a response time of 3 s, 0.051 μM as a detection limit at the two linear ranges from 0.15 to 1 mM and from 10^3^ to 16 10^3^ μM with sensitivities 0.301 μA μM^−1^ cm^−2^ and 0.133 μA μM^−1^ cm^−2^ respectively with good selectivity, stability, and reproducibility.

Polymers can be biocompatible, stable, and conductive and can be combined with several nanomaterials, enhancing their stability and conductivity. Based on the synergetic effect of polymer and other nanomaterials, the electrocatalytic efficiency of nanocomposites for nitrite and nitrate is improved. As a result, an increase of the concentration range for detection and the amelioration of the sensitivity and selectivity can be obtained.

### 3.4. Phosphorus Sensors

Table 6 summarizes the most recent trends in potentiometric and amperometric electrochemical sensors for phosphate detection underlining the new progress in developing nanomaterials and nanocomposites to fulfill the selectivity and sensitivity requirements.

#### 3.4.1. Polymeric Sensors

**Figure 7:**
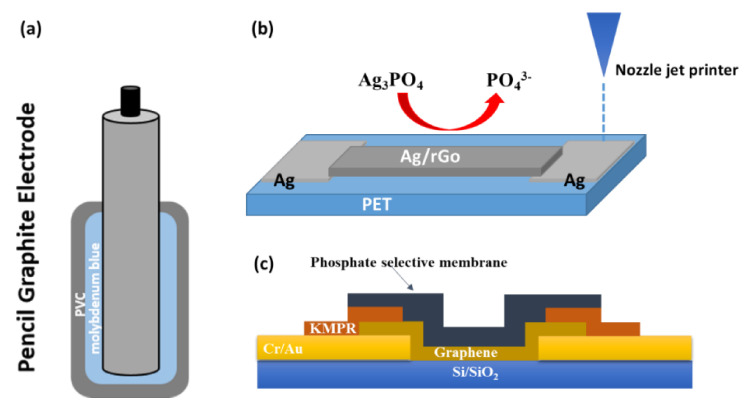
Redrawing of (**a**) Pencil graphite sensor coated with Molybdenum blue functionalized poly(vinyl chloride) following [212], (**b**) Nozzle-Jet-Printed Silver/Graphene Composite/Field-Effect Transistor Sensor following [212] and (**c**) ISFET structure with the phosphate selective membrane following [219].

Nanomaterial-doped conducting polymers are a unique category of composite materials that combine the advantageous properties of both nanomaterials and organic conductors. They have been applied in a wide range of uses, such as electrochemical sensing [211]. In this direction, some polymers showed their excellent role in phosphorus determination. A molybdenum blue-modified poly(vinyl chloride) layer electrodeposited onto a pencil graphite electrode was developed as a novel electrochemical sensor for phosphate determination. The use of polyvinyl chloride as a coating agent for the functionalized electrodes significantly improved the stability (see Figure 7.a). The prepared sensor showed a high sensitivity for phosphate ions with a low LOD and limit of quantification [212]. Also, a chitosan-smectite biocomposite was doped as an electroactive element in a PVC membrane potentiometric sensor for the direct and selective detection of monohydrogen phosphate ions. It exhibited good merits, including high sensitivity and stability, short response time, low detection limit and a broad linear detection range [213]. A gold phosphate electrode has been coated with polyaniline film and phosphate-doped polyaniline as an ion-selective membrane. Polyaniline was chosen based on its better conductivity over other polymers (such as polypyrrole, polythiophene, PVC) because of its low impedance. It achieved an excellent linear detection range and detection limit. High sensitivity and good stability are the major practical advantages of this phosphate sensor with a response time of fewer than 10 s. The drift value of the electrical voltage during 12h of continuous observation was 0.05 mV/h, with a lifetime of over 40 days. Thus, it is appropriate for either one-time or long-term in situ surveillance of industrial water, groundwater, river water, and natural seawater [214].

Polymers and especially conductive polymers are therefore nowadays considered suitable sensitive materials for preparing specific, accurate and reliable sensors. Also, the nanostructuring of electrodeposited polymers, as the electrosynthesis of polymeric nanowires or nanotubes, has considerably improved detection performance [212]. In this direction, future research will likely be dedicated to the development of new biosensors based on polymeric materials.

#### 3.4.2. Carbon Nanomaterials-Based Sensors

Carbon forms have been widely employed as an electrochemical sensing interface owing to their unique electrochemical properties. Graphene, carbon nanotubes, carbon nanofibers, and carbon dots are widely used in electroanalytical research for their chemical inertness, relatively wide potential window, small background current, and applicability to various types of essays. For example, other electrode materials, such as sputtered metal electrodes, have narrow potential windows and short lifetimes compared to carbon materials [229]. In the area of phosphate electrochemical sensors, carbon nanomaterials are frequently used. A cobalt-decorated graphene nanocomposite sensor has been developed for phosphate detection. The process consists of the electrodeposition of graphene oxide (GO) on a glassy carbon (GCE) electrode, then the electro-reduction to reduced graphene oxide (rGO), and finally, the electrosynthesis of cobalt nanoparticles on the GCE-RGO electrode. The potentiometric response for an acceptable detection range indicated a good linear relationship with the logarithm of the phosphate levels with a slope of −31.6 mV per decade of change in concentration [225]. Moreover, a phosphate sensor was elaborated based on screen-printed electrodes functionalized with carbon black nanoparticles. Amperometric measurements were performed by electrochemical reduction of the corresponding molybdophosphate complex. Carbon black nanoparticles showed the ability of quantification of the molybdophosphate complex at a low applied voltage. Under optimized conditions, a wide linear range was achieved, with a limit of detection of 0.1 mM. The device was evaluated in potable water, in rivers, in aquariums and in sewage water samples providing satisfactory recovery results in accordance with a spectrophotometric reference procedure that proved the suitability of this modified carbon black screen-printed electrode coupled with the use of molybdate to measure phosphate in aqueous samples [216]. A FET sensor based on nozzle-jet-printed Ag/rGO-composite on poly(ethylene terephthalate) (PET) substrates (see Figure 7b). An Ag precursor ink and Ag/rGO hybrid ink were used for the good adhesion of printing on the FET sensor. The sensor demonstrated high sensitivity, wide detection linear range and a very low detection limit. Also, it showed long-term stability, good selectivity toward interferences, high reproducibility, and applicability in different real water samples with high accuracy [217]. An ion-sensitive field-effect transistor with a hybrid graphene/ionophore membrane is fabricated (see Figure 7c). As a surrounding condition can easily alter the electrical behavior of the graphene, CVD graphene is chosen as the sensing material. Also, graphene has no selective capability for specific ions, and for that, a phosphate-selective membrane is to be used. The membrane is fabricated by a molecularly imprinted polymer (MIP). The sensor exhibited a detection limit of 1.79 μM and a response time of 10 s [219]. Implementation progress of carbon nanomaterials for electrochemical sensors opens the possibility of reliable, fast, easy, highly sensitive, selective and cost-effective determination of specific contaminants [221].

#### 3.4.3. Metal and Metal Complex-Based Sensors

Different metal-complex-based nanomaterials were used in the literature for phosphate sensing. Metallophthalocyanines (MPCs) are gaining more much interest in this direction. Due to their metal centers, MPCs are applied in chemical sensors, particularly for the detection of toxic ions [230]. An impedimetric electrochemical sensor based on new copper phthalocyanine derivative (copper phthalocyanine-C,C,C,C-tetracarboxylic acidpolyacrylamide) modified gold substrates has been elaborated. Under the optimized conditions in terms of polarization and frequency range, the developed sensor provided a large linear range with a very low LOD of 9.48 10^−11^ M and good sensitivity [221]. A capacitance chemical sensor-based silicon nitride substrate (AlCu/Si-p/SiO_2_/Si_3_N_4_) functionalized with copper C,C,C,C-tetracarboxylic phthalocyanine-acrylate polymer adduct (Cu(II)TCPc-PAA) was developed for phosphate ions determination. It showed good performance, with a Nernstian sensitivity of 27.7 mV/decade and a low LOD. The developed sensor showed a high selectivity against several interfering ions such as chloride, sulfate, carbonate and perchlorate. Thus, it is considered very promising for sensitive and rapid detection of phosphate [220]. As well as, as phosphate receptors transition metal ions Zn^2+^ and Cu^2+^-BPMP-doped in polymeric membranes (BPMB = 2,6-bis(bis(2-pyridylmethyl)aminomethyl)-4-methylphenol) have been also used. Zn^2+^ has high phosphate-binding stability, and Cu^2+^ has a strong anion binding behavior as their electrical configuration allows high ligand stabilization effects [222]. A properly aligned Pt/Au alloy nanowires network is synthesized and used successfully as a substrate to develop phosphate biosensors. Pyruvate oxidase was immobilized on the nanowire surface with co-factors using a cross-linking approach. As a result, the actual phosphate biosensor has high sensitivity and a wide linear range towards phosphate detection. In addition, it exhibits good selectivity and stability, promising high potential in phosphate detection applications [223].

### 3.5. Disinfectants and Byproducts

Ever since the guidelines were laid down for the DBPs by recognizing health issues, there has been a multitude of research on detecting these compounds in water. Several traditional techniques like gas, liquid and ion chromatography, mass spectroscopy and fluorescence spectroscopy have been used for their detection [231]. However, electrochemical sensors are attractive options because of the ease of use, portability, and cost-efficiency.

Based on carbon material composites, a CNT modified copper electrode with differential voltammetry measurements could detect ammonia with a linear range from 3 to 100 µM and LOD of 3.47 10^3^ μM [232]. A glassy carbon electrode modified with silver nanoparticles doped chitosan hydrogel film was used to detect trichloroacetic acid. The linear range of operation was 3 to 56 µM with LOD as 1.1 µM using Amperometry [233]. In another work, Titanate nanotubes were self-assembled onto a glassy carbon electrode modified before self-assembly with a chitosan membrane. Thionine was immobilized on the TNT/CTS/GCE surface, acting as an electrochemical probe to detect Trichloroacetic acid by cyclic voltammetry. The linear range of detection obtained was 15 µM to 1.5 10^3^ μM [234].

Metal-based sensors have been often used for the detection of DBPs by modifying electrodes with metallic particles because of the high catalytic nature of the particles. A pure silver-electrodes-based sensor was used for detecting three DBPs, namely trihalomethanes, bromoform and chloroform by stripping voltammetry [235]. Detection of brominated trihaloacetic acids based on voltammetry and chemometric analysis on a gold electrode was also demonstrated [236].

A voltammetry-based detection of trichloroacetic acid has been carried out by modifying the carbon ionic liquid-based electrode with palladium-graphene composite and then immobilizing hemoglobin. The electrode response was linear in the range from (160 × 10^−5^ to 130 × 10^−5^) μM and LOD of 500 μM [171]. An amperometric sensor based on a glassy carbon electrode modified with gold and silver nanorods was used for the detection of trichloroacetic acid with a linear range from 0.16 µM to 1.7 µM and LOD of 0.12 µM [237].

Metal-oxide based sensors such as iron oxide (Fe_3_O_4_)-modified carbon paste electrodes have been utilized to detect chlorite using square wave voltammetry. The LOD achieved was 0.0086 µM [238]. Fe_3_O_4_ was again used in another work on glassy carbon electrodes to detect trichloroacetic acid with a linear range from 70 to 205 µM [239]. A nickel oxide modified carbon ionic liquid electrode with hemoglobin immobilized on the electrode could detect trichloroacetic acid with LOD of 500 μM and linear range from 15,000 to 10 × 10^3^ μM [240]. In a similar work, the same electrode, but modified with tin oxide, could detect trichloroacetic acid with a LOD of 0.615 μM [241]. Myoglobin-based enzyme immobilization was demonstrated on carbon ionic liquid electrode modified with titanium oxide for the detection of trichloroacetic acid. The linear range achieved was from 5.3 to 114.3 × 10^3^ μM [242]. Table 7 provides the list of sensors developed for the detection of various DBPs.

Even though the research on the electrochemical detection of DBPs is significant, it is primarily focused only on detecting major DBPs according to the amount present in water. However, the other DBPS though present in low quantities, is still harmful even at low concentrations. The detection is done using traditional methodologies like colorimetric [251], solid phase extraction, and gas chromatography [252]. The use of redox proteins as a catalyst for detection also has its disadvantages like high fabrication cost, denaturation of the proteins, reliability issues of the sensor. Furthermore, no papers have shown an in-situ application of the sensors. As there is a high chance of forming multiple disinfectant products simultaneously, a careful and systematic study on the selectivity of the sensors is not performed in some of the papers. Hence, there is a dire need to develop electrochemical sensors selective and sensitive to all the DBPs, which are potentially harmful to humans and the environment.

### 3.6. Emergent Contaminant Sensors

Polyphenolic compounds (PCs) have received exceptional attention due to their harmful effect on the human body and the environment. Polyphenols are omnipresent secondary metabolites in foods [62,253]. They contain phenolic hydroxyl group(s), which are the basis of their antioxidant activity. In general, the reaction of this antioxidant takes place with the loss of one electron in order to get a non-toxic, stable composition unable to propagate the reaction [63]. Antioxidants undergo many interactions in the food matrix, like, preventing fat necrosis and reduce the harmful effects of nitrogen and active oxygen [63]. Several works have been made to introduce sensitive and straightforward methods to evaluate antioxidant capacity (AOC) and quantify polyphenols in food. Table 8 summarizes many sensors for some emergent contaminants. 

#### 3.6.1. Carbon-Based Materials for Phenolic Compounds Detection

Carbon materials are not only good electronic conductors, cheap, abundant and easy to work with and chemically inert, but also suitable for making composites, as can be already concluded from the previous discussion. Defects and functional groups in carbon materials, inherently present or intentionally introduced, can improve the electrochemical sensor response. Materials traditionally used for the electrochemical detection of phenolic compounds are graphene, carbon nanotubes, carbon black and carbon paste-based hybrid electrodes as an alternative strategy [62].

**Figure 8:**
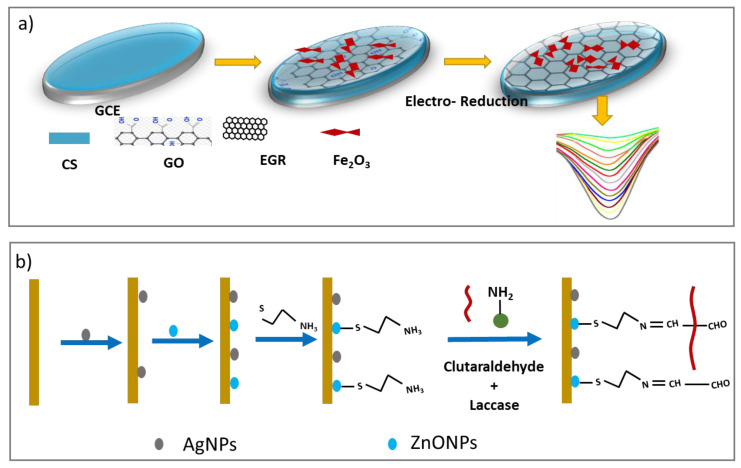
Redrawing of (**a**) GCE-GR reduced-Fe_2_O_3_/Chitosan following reference [255], and (**b**) Chemical sequence of electro polymerization of AgNPs/ZnONPs onto Au electrode and chemical reaction of immobilization of enzyme onto modified electrode following reference [286].

Undoubtedly, graphene is one of the most important discoveries of the last decade in terms of nanomaterials. This two-dimensional material opened new gates in the electrochemical sensing application. A nanocomposite of graphene/poly (3,4-ethylenedioxythiophene):poly(styrene sulfonate) electrosprayed on a modified screen-printed carbon electrode (SPE-GR/PEDOT/PSS) [254]. The nanocomposite elaboration was optimized and characterized physically and electrochemically to improve the active surface area and charge transfer kinetics. The sensor has been used to quantify the antioxidant capacity of 2,2-diphenyl-1-picrylhydrazy radical (DPPH). The developed electrochemical approach displays a LOD of 0.59 μM at the range from 5 to 30 μM. It was tested in real samples of herbal beverages and Thai herbs and compared with the spectrophotometric method. No variation was obtained between the two methods. A glassy carbon electrode modified with a hybrid material based on chitosan (CS), fishbone-shaped Fe_2_O_3_ and reduced graphene oxide (GCE-GR reduced- Fe_2_O_3_/Chit) was used to quantify gallic acid as a phenolic compound (Figure 8a) and to estimate the antioxidant capacity index of them [255]. The results show excellent linearity of the current versus the log of the concentration at a wide range from 1 μM to 0.1 mM and a LOD of 0.15 μM. The GCE-GR reduced- Fe_2_O_3_/Chit electrode was used to determine polyphenols in wine as a real sample.

Several reports introduce carbon nanotubes as sensitive material for the electrochemical detection of phenolic compounds. A simple electrochemical sensor has been proposed by Lismery et al. [256] to detect gallic-acid based on carbon electrodes (GCE) modified with single-walled carbon nanotubes (SWCNTs). After modifying glassy carbon electrodes with SWCNTs, the results suggest an improvement of anodic current and electron-transfer kinetics due to its superior characteristics. After optimizing DPV measurements, a detection limit of 0.3 μM at the concentration range from 0.5 μM to 15 μM of gallic acid was achieved. Based on the calibration curve of gallic acid, traces of polyphenols were estimated in wine with FC (R = 0.980).

Another sensor based on MWCNTs has been reported by Arribas et al. [284], who proposed a strategy for quantifying polyphenols in wine. GCE was modified via drop-casting of different nafion or polyethylenimine (PEI) CNTs dispersions. Then various polyphenols were evaluated (gallic acid, caffeic acid, ferulic acid, *p*-coumaric acid), resulting in an improvement of their oxidation on the surface of the MWCNTs modified electrodes. The response was linear for the four model analytes in the concentration range from 0.1 μM to 100 μM, and the limit of detection (LOD) using the two detection potentials was below 0.1 μM.

Li et al. proposed a susceptible sensor to quantify the BPA involving the use of GCE functionalized with COOH-MWCNTs/GCE) [274]. The presence of COOH group at the surface of the MWCNT improves the current oxidation of COOH-MWCNT/GCE compared to the bare GCE and the MWCNT/GCE. At a pH of 7, a sharp oxidation peak was observed at 550 mV in LS voltammograms. In the concentration range from 10 to 104 × 10^−3^ µM, a linear and a LOD of 5 × 10^−3^ µM are shown. The sensor has been demonstrated to be suitable for the effective detection of BPA in real samples.

Rather et al. presented an ultrasensitive electrochemical sensor to quantify the BPA based on fullerene [285]. In comparison to GCE, the sensor shows high electrocatalytic activities, lowering anodic overpotential and creating a significant increase in the BPA anodic current, according to their analysis. Rather et al. also calculated a variety of kinetic factors, including electron transfer number (n), electrode surface area (A), diffusion coefficient (D), and charge transfer coefficient (α). The oxidation peak current displayed a linear relationship in a concentration range from 0.074 to 0.23 µM, with a LOD of 0.37 × 10^−2^ µM, under optimal conditions. The sensor performance was validated by detecting BPA in wastewater samples and promising analytical results for identifying BPA at trace levels were registered.

#### 3.6.2. Metallic Nanomaterials

As can be concluded from the previous discussion, metallic nanomaterials possess intrinsic and stable activity making them suitable for the functionalization of different electrodes as a sensitive material to quantify phenolic compounds.

An electrochemical sensor has been developed by Sheetal et al. [286] based on laccase covalently onto nanocomposite of (AgNPs) and (ZnONPs) modified gold (Au) electrode surface using electrodeposition of AgNPs/ZnONPs/Au (see Figure 8b). The AgNPs/ZnONPs/Au electrode was used to detect phenolic compounds in wine samples. The amperometric measurements show a sensitivity of 0.71 μA μM^−1^ cm^−2^ for the guaiacol phenol, a limit of detection equal to 0.05 μM and a wide linear range from 0.1 to 500 μM.

Another possibility is to employ copper oxide nanoparticles (CuO NPs) as a sensitive layer for detecting phenolic compounds. Pino et al. [287] demonstrated the use of CuO NPs drop-casted onto screen-printed carbon electrodes to detect catechol, phenol, and 4- dichlorophenol with ultra-sensitivity and detection limits of 0.047 µM 0.5 to 2.5 µM at the range from 0.5 to 2.5 µM. In this approach, in the presence of phenols, the oxidation and reduction current of copper decrease due to the formation of a complex between catechol and Co-NPs.

A glassy carbon electrode (GCE) loaded with Ag nanoparticles and polyguanine has been proposed for detecting bisphenol A (BPA) (Ag-PGA) by Hong et al. [288]. The electrode, modified using a simple one-step procedure, has a considerably increased oxidation peak current corresponding to BPA due to its great adsorption capacity, resulting in a larger linear range from 0.01 to 100 µM, and a low LOD of 10^−3^ µM (S/N = 3). The approach has been successfully validated for water samples.

In another study, Chen et al. succeeded in elaborating nano-dendrites at the surface of GCE by one-step electrodeposition in the presence of a gold precursor AuCl4^−^ and L-asparagine [289]. The electrodeposited nano-dendrites were functionalized with 4-mercaptobenzoic acid (4-MBA), leading to better catalytic performance and sensitive and selective detection of BPA using DPV. The proposed sensor shows an oxidation current peak at around 514 mV BPA, which increases linearly by increasing the BPA concentrations from 0.05 to 55.0 μM (R_2_ = 0.995) and a LOD of 12 10^−2^ µM. Finally, the sensor was utilized to determine trace quantities of BPA in spiked samples and has shown satisfactory results.

#### 3.6.3. Polymeric Sensors

Conducting polymers have been hailed as potential electrocatalytic materials with substantial advantages for BPA electrochemical sensors [290]. Several methods have been proposed for the functionalization of the electrode with polymers, such as electrochemical polymerization solvent evaporation, dip and spin coating, radiofrequency plasma discharge, etc. Poly(3,4-ethylenedioxythiophene) (PEDOT) and polyaniline (PANI) are considered essential materials because of their regular and organized chemical structure, as well as high stability and conductivity [291].

Mazzotta et al. examined the electrochemical behavior of BPA over PEDOT-modified GCEs via CV [292]. BPA oxidation generated a BPA polymer (pBPA) with excellent redox activities, with cathodic and anodic peaks at 0.01 and 0.15 V, respectively. Therefore, they approximated the content of the deposited pBPA by electrochemical and spectroscopic analyses via X-ray photoelectron spectroscopy (XPS). The effects of the scan rate and pH on pBPA film oxidation behaviors have been investigated. According to the studies, the oxidation current has a linear behavior in the range from 90 to 410 μM, with a LOD of 55 μM. Consequently, the amperometric BPA determination outputs were gathered with a repetitive potential step program to give a linear response to BPA in a concentration range of 40 to 410 μM, with a LOD equal to 22 μM and sensitivity equal to 1.57 μM^−1^ cm^−2^. The sensor exhibited acceptable features of reproducibility and anti-interference, showing a successful application for the detection of BPA in mineral water samples.

Poorahong, et al. proposed a simple and sensitive amperometric sensor to quantify BPA based on polyaniline nanorods and MWCNT in a pencil graphite electrodes [293]. The results show, when compared to the original pencil graphite electrode, the functionalized electrodes had higher electroactivity for BPA oxidation. The sensor shows a linear response to BPA in the 1.0 to 400 μM concentration range under optimal experiments and a LOD equal to 10 × 10^−2^ µM. At 100 M of BPA, the modified electrode has a remarkably stable response, allowing for up to 95 injections with a relative standard deviation of 4.2%. Boiling water spiked with BPA from four brands of baby bottles yielded recoveries ranging from 86% to 102%.

#### 3.6.4. Dihydroxybenzene Isomers

Dihydroxybenzene has three isomers: hydroquinone (HQ), catechol (CC), and resorcinol (RC) (DHB). The DHB is commonly used in industrial manufacturing, and traces of these toxic compounds can be found in the environment, especially in water supplies [294]. The three DHB isomers normally coexist as toxins in environmental water samples. If DHB isomers are released into the atmosphere, they can cause severe health problems [295]. Large amounts of DHB isomers can cause illnesses including kidney failure, tachycardia, cancer, and even death. Since the bare working electrodes have a low oxidation/reduction current reaction of these DHB compounds for the electrochemical sensing methods, it isn’t easy to distinguish the two or three DHB isomers. As a result, it is often important to decorate certain nanosized substances to create a new successful functioning electrode. Carbon-based nano-sized materials have been hailed as promising service materials for various applications, including sensors [296]. Table 9 provides an overview of electrochemical sensors for dihydroxybenzene isomers.

(a) Carbon-based hybrid nanocomposites

Experiments have shown that well-aligned hybrid structures of graphene and carbon nanotube can be formed. Their mechanical properties are easily tunable due to their highly tailorable structures hybrid structure of CNT and Gr, one of the most promising carbon derivatives. Carbon nanotubes were sandwiched between graphene sheets that served as spacers and provided diffusion paths for smooth and rapid ion conduction in the CNT-GR composite with a highly porous structure.

For the quantification of trace DHB isomers, Yang and Weikun [297] prepared hybrid MWCNTs in GO-cetyl trimethylammonium bromide (CTAB) composites adjusted GCE by Cyclic voltammetry (CV). CTAB/CNTs are negatively and positively charged, respectively, despite they are not neutral materials. As a result, there could be a close relationship between each surfactant and CNTs. CT, HQ, and RC sensor calibration curves were obtained in the range of 0.1 to 400 µM, 0.1 to 200 µM and 1 to 100. The detection limits were 0.01, 0.03 and 0.1 µM for CT, HQ and RC, respectively. The CTAB-GO/MWNT sensor succeeded in detecting DHB isomers in tap water samples with promising results.

In their work, Yang et al. [299] presented a sensor based on MWCNTs coupled with RGONR modified GCE MWCNT@rGONR/GCE for simultaneous determination of HQ, CC and RC by DPV. The hydrothermal process was used to make the reduced graphene oxide nanoribbon (RGONR) composite in this analysis. For HQ, CC, and RC, the detection limit is calculated to be 0.8 µM, 0.95 µM and 0.1 µM respectively, at the concentration range from 2 to 80 μM. The findings showed that even after a month of storage, the changed electrode retained 95.1% of the original HQ current. In the end, the MWCNT@rGONR/GCE showed a satisfactory result toward the detection of HQ, CC, and RC simultaneously in water samples.

(b) Carbon material-supported bimetallic composites

Au@Pd nanoparticles can be anchored to the surface of RGO sheets as signal amplifiers, amplifying the catalytic oxidation peak currents. Chen et al. [306] reported the simultaneous quantification of DHB isomers using Au@Pd nanoflower (PdNF)-RGO (Au@PdNF-RGO). The Au@PdNF-RGO-modified GCE produced combined RGO’s specific conductivity with the Au@Pd’s superior catalytic efficiency. The Au@PdNFs/RGO was deposited onto the GCE using CV in this study. The combination of Au@Pd’s outstanding electrocatalytic properties and RGO’s excellent conductivity produced excellent results. In contrast to AuNPs/RGO-GCE and RGO/GCE, the updated electrodes showed a high sensitivity for identifying three target isomers with good operation. The LODs of the sensor were 0.5 μM, 0.8 μM and 0.7 μM, respectively for HQ, CC and RC.

Wag et al. [306] also presented a sensor based on a Au@Pd/RGO nanohybrid-modified GCE (Au@Pd/RGO/GCE) detection of HQ and CC with a LODs of 0.01 μM and 0.1 μM, respectively. The electrochemical activity of the Au@Pd/RGO modified electrode was observed to be higher than that of the electrochemically deposited Au@PdNF/RGO at the surface of GCE. As a result, the Au@Pd/RGO composite electrode has a broad electrochemical surface area, which results in high electrocatalytic activity. It thus has an extensive range of concentration, 0.01to 400 μM for HQ and 0.1 to 400 μM for CC).

(c) Carbon material-supported conducting polymers

The integration of conductive polymer with CNT or Gr as a composite exhibited a synergetic effect, leading to the augmentation of the electronic and mechanical characteristics of the constituent components [307].

Jiang et al. [308] investigated the electrochemical capability of the poly-tryptophan-functionalized Gr (p-Trp-Gr) toward the oxidation of HQ and CC. The results show that the involvement of Trp has an indole conjugate structure, and it assures the dispersion of Gr via the p–p interactions. Also, in PBS at pH 7.0, Trp on Gr could speed up the electron transfer rate of the isomers and provides a detection limit of 0.221 and 0.086 μM for HQ and CC, respectively.

Song et al. [309] reported a composite based on a poly(diallyl-dimethylammonium chloride) (PDDA)/MWCNTs/Gr-PDDA/MWCNTs/Gr-modified GCE, for simultaneous detection of HQ and CC. PDDA (an ordinary and water-soluble cationic polyelectrolyte) was employed as the covalent cross-linking agent to bind MWCNTs and GR. The electrostatic activity allows the positively charged PDDA colloid to be easily coated on the negatively charged Gr or MWCNTs surfaces in this process. The results revealed that the composite MWCNTs-PDDA-GR successfully presented an enhanced electron transfer and a high electroactivity toward the HQ and CC oxidation. The proposed sensor (PDDA)/MWCNTs/Gr-PDDA/MWCNTs/Gr/GCE showed a range of concentration for both targets HQ and CC from 0.5 to 400 μM with detection limits (S/N = 3) of 0.02 and 0.018 μM, respectively and a successful detection in water samples.

#### 3.6.5. Further Emergent Contaminants

Most of such substances occur in the environment and are persistent to a longer extent. Common contaminants could be either described as highly persistent and do not bio-degrade in the environment such as heavy metals, or persistent and slowly biodegradable, which is the case of numerous pharmaceuticals (e.g., carbamazepine). Also, persistent substances that are water-soluble can get easily into water and chemicals that may not be persistent and can be converted or removed by natural processes.

Improvements in analytical chemistry have made the detection of these pollutants possible, even at trace levels. The scope of this review is to present an overview of the most relevant electrochemical detection sensors of these contaminants, which are summarized in Table 2. An electrochemical immunosensor based on hexestrol (HEX)-2-aminoethanethiol hydrochloride (AET)-gold nanoparticles (Au NPs)-glassy carbon electrode (GCE) was developed for simultaneous determination of four different phenolic estrogens: HEX, diethylstilbestrol (DES), dienestrol (DE) and bisphenol A (BPA). DES > DE > BPA > HEX was the amperometric response sequence by differential pulse voltammetry (DPV), with detection limits of 0.0045, 0.0027, 0.0036 and 0.0045 µM, respectively (S/N = 3). The results show a good linear range and selectivity and satisfactory accuracy in real samples for DE detection [268].

Long-term exposure to 17β-estradiol (E2), at even very low levels, can damage the endocrine system and cause cancer. For that reason different electrochemical sensors based on various nanomaterials have been developed. A simple aptasensor based on split DNA aptamers for E2 was used. When E2 is present, the split aptamers are bound to E2 and form the complex split1-E2-split2 on the electrode surface. The sensor recognizes E2 within 30 min with a wide linear range and detection limits of 0.5 pM and 0.7 pM in tap water and milk, respectively [269]. A label-free integrated microfluidic paper-based analytical device was also fabricated for the detection of 17β-E2. Multi-walled carbon nanotubes/thionine/gold nanoparticles (AuNPs) nano composites were synthesized and coated on a screen-printed working electrode (SPWE) to immobilize the anti-E2. The sensor can detect 17β-E2 as low as 10 pg/mL with a wide linear range (2 × 10^−3^–1.79 µM) [271].

For pyrethroid insecticide detection, a polished silver solid amalgam electrode (p-AgSAE) can be used. The proposed sensor was applied in natural water and tea samples and showed high robustness, good stability and sensitivity [272]. A nano-cylindrical strontium titanate/N-doped graphene (SrTiO_3_/N-GNS) hybrid composite-based sensor was reported for simultaneous detection of diphenhydramine (DPH) and bromhexine (BRO). The sensor showed a wide linear range from 0.038 to 100 10^3^ µM for DPH and 0.03 to 90 10^3^ µM for BRO with detection limits of 2.1 and 1.9 10^−3^ µM, respectively [273].

## 4. Conclusions and Perspectives

This review focuses on electrochemical sensors for pesticides, nitrate, nitrite, phosphate, water hardness, disinfectant, and some emergent contaminants. Within the last five years, most of the reviewed sensors show suitability for real applications from the point of view of sensitivity and interference tests. The combination of electrochemical sensors with novel nanomaterials enables the efficient detection of several unknown and unquantified contaminants. Functionalization by nanomaterials can be in the form of a composite such as carbon nanomaterial with metallic nanoparticles. Several cases have demonstrated that nanomaterials-based label-free electrochemical sensors can realize a high sensitivity towards specific analytes with excellent selectivity. However, a plethora of research in electrochemical sensors keeps on improving sensor’s sensitivity and selectivity.

Nevertheless, electrochemical sensors are generally not specific as some compounds, which can undergo electrochemical transformation within an analysis potential window, can interfere with the analyte under investigation. This could be at the same time advantageous for multiple ions/molecules detection, which can be achieved by simultaneous detection when there is a significant oxidation peak potential separation between the different species. In some cases, several compounds can be detected at the same potential, such as pesticides of the same group.

Electrochemical sensors are primarily demonstrated in a controlled lab environment. Only a few sensors available today are in actual use for on-site or in-situ measurements. This is still challenging because of different requirements of sensor properties, such as repeatability, reproducibility, and stability. The same situation occurs with sensors based on nanomaterials that not often were demonstrated for use in natural water, such as in sea water. Thus, there are a few perspectives and remaining challenges in this field, including:

- A lack of electrochemical sensors for in-situ applications [310].
- Real-time stability and reusability.
- Large-scale and inexpensive fabrication.

Developing efficient strategies to overcome these challenges is required for EC sensors to be commercially competitive for in-situ monitoring. Different types of devices were developed for pesticide detection, trying to overcome the generally poor selectivity of electrochemical methods. This issue arises from the fundamental properties of electrochemical processes. The strategy for resolving it was found in the specific interactions between the sensor and the analyte (pesticide). One of the most promising routes to enhance detection selectivity is biomimetic sensors that reach excellent sensitivity comparable to high-performance liquid chromatography and other advanced chromatographic methods. However, the problem with any sensor relying on specific guest-host interactions is that it can detect only one or a minimal number of analytes. We anticipate further developments in biomimetic sensor arrays combined with machine learning, artificial intelligence, and cloud computing. The combination of EC detection and A.I. with optical spectroscopy such as SERS will allow overcoming fundamental limitations inherent to those detection methods working alone. For example, the quantification capabilities of EC sensors with the label-free selectivity of SERS often gives complex voltammograms and spectra from real-life samples, and machine learning algorithms could help making sense of it all. Nevertheless, forecasting when small and portable solutions for routine on-field applications will become available is an exciting task that still elude us.

## Figures and Tables

**Table 1:** Maximum allowable concentration for water quality assessment for EU [19], WHO [20] and USA-EPA [21].

Analyte	EU (mg/L)	WHO (mg/L)	USA-EPA [mg/L]
Nitrate	50	50	10
Nitrite	0.50	3	1
Phosphate	-	5	-
Ammonium	0.2	1.5 at Alkaline pH	-
Chlorate	0.250	0.7	-
1,2-dichloroethane	0.003	0.03	0.04
Epichlorohydrin	0.00010	0.0004	0.3
Trihalomethanes (total)	0.1	0.1	0.08
Haloacetic acids (HAA5)	0.06	-	0.06
Halogenated acetonitriles	-	0.02	-
Trichlroacetaldehyde	-	0.1	-
2,4,6-Trichlorophenols	-	0.2	0.3
Bisphenol A	0.0025		2.5
Pesticides	0.0001	0.00003–0.2 *	
Total pesticides	0.0005		
Calcium	-	10–500	-
Magnesium	-	52.1	-

* According to the type.

**Table 2:** Overview of electrochemical sensors for pesticides, based on (modified) carbon materials at the working electrode.

Detection Method	Materials	Analyte	LOD (µM)	Dynamic Range (µM)	Comments	Reference
SWV *	BDD	Parathion	0.043	-	Low interference with organic pollutants compared to HMDE *	[90]
SWV	BDD	Atrazine	0.01	0.05–40	Good selectivity and repeatability	[91]
SWV DPV *	BDD	Methomyl	19 1.2	66–420 5.0–410.0	Good recovery with real samples	[92]
adsorptive stripping SWV	Sol-gel carbon ceramic electrode	Fenitrothion	0.0016	5000–1,000,000.1–50	Demonstrated on-site monitoring	[93]
SWV	Graphite-modified basal plane pyrolytic graphite electrode	Methyl parathion	3	79.0–263.3	Applied for drinking water	[94]
adsorptive stripping SWV	Poly(4-amino-3-hydroxynaphthalene Sulfonic acid) modified GCE	Fenitrothion	0.7 × 10^−3^	0.001–6.6	Good recovery in spiked water samples	[95]
SWV	Sarbon black modified GCE	Mesotrione	0.026	0.040–7.2	Applied for real water samples and juice	[96]
cyclic voltammetry and SWV	Peptide nanotubes on modified pencil graphite electrode	Fenitrothion	0.0196	0.114–1.712	Good recovery in spiked water samples	[97]
DP adsorptive cathodic stripping voltammetric	Single-walled carbon nanohorns and zein modified GCE	Fenitrothion	0.012	0.99–12	Good repeatability and reproducibility applied for real water samples and juice	[98]
SWV	Screen-printed carbon electrode	Bentazone	0.034	-	Analysis time 10 s, reusable at least 15 times, sensitivity of 0.0987 × 10^6^ μA/M	[99]
adsorptive stripping voltammetry	Nano poly(3-methyl thiophene)/multiwalled carbon nanotubes	Isoproturon, Voltage cypermethrin Deltamethrin fenvalerate Dicofol	26–100	1.43–4.47 1.45–4.47 0.26–4.47 0.34–4.47 1.09–4.47 0.967–4.47	Good recovery in spiked water samples	[100]
DPV	Multiwalled carbon nanotubes-poly(acrylamide) nanocomposite	Methyl parathion	0.002	0.005–10	Demonstrated for environmental water samples	[101]
SWV	Graphene-based electrochemical sensor	Isoproturon:	20	20–1000	Demonstrated for water, soil and vegetable samples	[102]
DPV	Graphene quantum dots with oxime as electroactive probe	Fenthion	6.8 × 10^−6^	1.0 × 10^−5^–5.0 × 10^−2^	Performance demonstrated for water and soil samples	[103]
DPV	Ionic liquid–graphene nanosheets	Methyl parathion	1.9 × 10^−5^	0.09–0.04	Satisfactory stability and reproducibility demonstrated for spiked water samples	[104]
DPV	N-methyl-2-pyrrolidone exfoliated graphene	Carbendazim	0.78 M	0.005–1.57 × 10^−6^ M	Demonstrated for ground water, soil, and cucumber samples	[105]
DPV	Pillar[5]arene/reduced graphene nanocomposite	Methyl parathion	3 × 10^−10^ M	0.001–150 × 10^−6^ M	Demonstrated for soil and waste water samples	[106]
DPV	Cellulose microfiber entrapped reduced graphene oxide	Fenitrothion	0.008	Linear range up to 1134	Demonstrated for different water samples	[107]

* SWV: Square wave voltammetry, DPV: Differential pulse voltammetry, HMDE: Hanging mercury drop electrode.

**Table 3:** Overview of electrochemical sensors for pesticides, based on molecularly imprinted polymers.

Detection Method	Materials	Analyte	LOD (µM)	Dynamic Range (µM)	Comments	Reference
CV	Molecularly imprinted polypyrrole membrane	2,4-Dichloro-phenoxy acetic acid	0.83	1.0–10	Successful determination in real samples	[109]
photoelectrochemical technique	Polypyrrole-based MIP composite with TiO_2_ *	2,4-Dichloro-phenoxyacetic acid	0.01	-	Demonstrated for spiked water samples	[110]
capacitive sensor	Polyquercetin-polyresorcinol-Gold nanoparticles by MIP technique	Methyl parathion	3.4 × 10^−4^	0.07–1	Good recovery and low interference in water samples	[111]
impedimetric	MIP/sol–gel, different monomers	Methidathion			Proof-of-concept experiments	[112]
cyclic voltammetry and SWV	MIP suspension polymerization, modification of carbon paste electrode	Diazinon	7.9 × 10^−5^	2.5 × 10^−3^–0.10.1–2.0	Good recovery in water and apple samples	[113]
potentiometric	Methacrylic acid (functional monomer), ethylene glycol dimethyl acrylate (cross-linker)	Endosulfan	20	20 to 12 × 10^−3^	Nernstian response, good stability	[114]
DPV	Methacrylic acid, ethylene glycol dimethacrylate and carbon nanotubes	Diazinon	1.3 × 10^−4^	5 × 10^−4^–1	No sample preparation for human urine, tap, and river water samples	[115]
SWV	MIP with carbon nanotubes	Dicloran	4.8 × 10^−4^	1 × 10^−3^–1	No interference	[116]
cyclic voltammetry	MIP from methacrylic acid, ethylene glycol dimethacrylate	Atrazine	716.26		Proof-of-concept, effects of ethanol solution analyzed	[117]
cyclic voltammetry	MIP/graphene oxide modified glassy carbon electrode	Profenofos	5 × 10^−3^	0.05–3500	Stable; enhanced selectivity vs. other structurally similar pesticides	[118]
DPV and EIS*	Acrylamide based MIP on gold electrode	Malathion	1.79 × 10^−7^	1 × 10^−7^–0.017	Good recovery in olive oil and fruit samples	[119]

* EIS: Electrochemical impedance spectroscopy.

**Table 4:** Overview of electrochemical sensors for water hardness ions in water.

Detection Technique	Electrode/ Materials	Analyte	LOD (µM)	Detection Range (µM)	Comments	Reference
CV, EIS	Screen-printed SPEs/carbon ink	Ca^2+^	1.0	10–10 × 10^4^	Detection in seawater	[160]
Potentiometric, EIS	Solid-contact ISE (SC-ISEs)	Ca^2+^	0.6	1–10 × 10^4^	Mineral water and tap water	[162]
Potentiometric	Glassy Carbon Electrode/Ca^2+^-ISM	Ca^2+^	0.16	0.3–1000	Diluted artificial seawater	[163]
EIS	MWCNTs/PDMS	Ca^2+^ and Mg^2+^	-	29.41–5882.35	In water bodies	[164]
EIS Potentiometric	Ionophores ISEs	Ca^2+^ Mg^2+^	100 100		In artificial fish-breeding water	[165]
SWASV *	MEMS-Based sensor on top of a silicon wafer	Ca^2+^ and Mg^2+^	29.41	294.1–1470.59	-	[166]
Amperometric	On-chip amperometric sensor with ion exchange membrane	Mg^2+^	5	-	-	[167]

* SWASV: Square wave anodic stripping voltammetry.

**Table 5:** Sensor for nitrogen ions (NO_3_, NO_2_).

Detection Principle	Electrode/Materials	Analytes	LOD (µM)	Dynamic Range (µM)	Comments	Refrerence
**Chronoamperometry**	Co_3_O_4_/RGO*/GCE^+^	Nitrite	0.14	1–380	Tap water. Recovery: 99.3–101.5%.	[168]
DPV	PEDOT-Gr*/Ta	Nitrite	7	20–2000 M	PBS. (RSD) 50 continuous CV cycles 4.5%.	[169]
DPV	GNPs/graphene/MCE	Nitrite	0.1	0.3–720	Lake water, river water, industrial sewage, and milk. Recovery: 96.0–103%.	[170]
CV *	Nafion/Hb *-Pd-GR */CILE	Nitrite	0.2	40–500	Tap water and Medical facial peel. Recovery: 96.17–101.24%	[171]
SWV	Cu/MWCNT */rGO/GCE	Nitrate Nitrite	0.02 0.03	0.1–75	Tap and mineral waters, sausages, salami, and cheese samples. Recovery: 98.3–102.5%	[172]
Amperometry	PANI@GO/GCE	Nitrite	0.5	0.002–44	Phosphate buffer	[173]
Amperometry	Ferrocene/rGO/SPCE *	Nitrite	0.35	2.5–1450	Spiked mineral water. Recovery: 95% and 101%	[174]
Amperometry	Ag/Cu/MWNT/GCE	Nitrite	0.2	1–1000	Lake water, Drinking water and Seawater. Recovery: 92–105%	[175]
DPV	Au Cu NCNs/GCE	Nitrite	0.2	10–4000	After storing in a refrigerator at 4 °C for 35 days, the peak current responses were still retained 98.60% of the initial values	[176]
Amperometry	GO-CS-AuNPs/GCE	Nitrite	0.3	0.9–18.9	Phosphate buffer	[177]
Amperometry	AuNPs-Fe_2_O_3_/FTO	Nitrite	0.07	1–1000	Tap and rain water samples	[178]
LSV *	3D lamellar nanocomposite/AgNS */rGO/β-cyclodextrin/SPCE	Nitrite	0.24	1–2000	Nitrite in spiked pickles.(RSD) = 2.35% (n = 5)	[179]
DPV	Fe_3_O_4_/GO/COOH/GCE	Nitrite	0.37	1–85 and 90–600	Phosphate buffer	[180]
SWV	LIG/f-MWCNT-AuNPs	Nitrite	0.9	10–140	Tap water	[181]
Amperometry	Co_3_O_4_-rGO/CNTs/GCE	Nitrite	0.016	8000–56,000	Recoveries: 95.7–102.2% 83.3% of initial sensitivity after one month storage	[182]
CV *	3D Au-rGO/FTO	Nitrite	-	20.99–5740	Phosphate buffer	[183]
Amperometry	Pt/Ni(OH)_2_/MWCNTs/GCE	Nitrite	0.13	0.4–5670	Milk Recoveries 96–104%	[184]
Amperometry	PANI/CNTs/GCE	Nitrite	1.6	-	PBS RSD 3.4% (n = 9)	[185]
Amperometry	Ni_7_S_6_/MWCNTs/GCE	Nitrite	0.3	1.0–4002	Lake later, Tape and water Pickle water	[186]
DPV	GO–MWCNT–PMA *–Au/GCE	Nitrite	0.67	2–10,000	Water RSD (n = 5) 4%	[187]
DPV	AuNPs-S-Gr/GCE	Nitrite	0.003	12.5–680.92	Water RSD (n = 3) 0.87%	[188]
Amperometry	rGO/Acr paper	Nitrite	0.12	0.40–3600	Milk and water	[189]
DPV	Self-assembled graphene CuNP/AuE	Nitrate	7.98	10–90	Lake water	[190]
Amperometry	CNT/PPy * film electrode with Nitrate reductase	Nitrate	170	440–1450	Nitrate in water	[191]
CV	Cu, Zn (SOD1 *) and nitrate reductase (NaR) coimmobilized on CNT–PPy modified Pt * electrode (NaR–SOD1–CNT–PPy–Pt)	Nitrate Nitrite	0.2 0.05	0.5–10,000 0.0001–1000	Human plasma, whole blood and saliva samples	[192]
CV	Ag-doped zeolite-expanded graphite-epoxy electrode	Nitrate	100	1000–10,000	Spiked tap water	[193]
SWV	Ag dendritic nanostructure on Au microelectrode array	Nitrate Nitrite	2	2–1000	River and lake water	[194]
CV	Cu^*^ nanoclusters, electrodeposited on Pt microelectrode	Nitrate	-	12.5–300	Fresh water	[195]
SWV	Cu microelectrode array	Nitrate	1.8	10–1070	Mineral water	[196]
LSV	Cu nanowire array	Nitrate	1.7–3	10–400	Mineral water	[197]
DPV	Cu microspheres decorated on polyaniline on microneedle	Nitrate	8	20–6000	Pre-treated river water	[198]
CV	Ag branchlike on Ag or carbon ultramicroelectrodes	Nitrate	3.2–5.1	4–1000	Synthetic aquifer	[199]

* AgNS: Silver nanostructures, Gr: Graphene, Hb: hemoglobin, GNP: graphene nanoparticles, SPE: Screen printed electrode, PPy: polypyrrole, PMA: 1-pyrenemethylamine, MWCNT: Multi walled carbon nanotubes, NPs: Nanoparticles, RGO: Reduced graphene oxide, GO: Graphee oxide, GCE: Glassy Carbon electrode, NRs: Nanoroads, SOD1: Zinc superoxide dismutase, CV: Cyclic voltammetry, LSV: linear sweep voltammetry.

**Table 6:** Overview about nanomaterials-based sensors for phosphate ion detection.

Method	Electrode/Materials	Analyte	Linear Range (µM)	LOD (µM)	Comments	Ref
Voltammetry (DPV)	molybdenum blue modified PVC*/pencil graphite electrode	K_2_HPO_4_	-	0.021	Measurement in soil sample	[212]
Potentiometry	Chitosan-clay/PVC	K_2_HPO_4_	1–10^4^	0.6	-	[213]
Chronoamperometry	Doped PANI*/gold electrode	KH_2_PO_4_	1–100	1	Response time < 1 s, electrode lifetime > 40 days in solution	[214]
Potentiometry	Graphene nanocomposite/Co microelectrode	KH_2_PO_4_	0.1–1	0.01	Effective measurement in lake water and sediment samples for soluble phosphorus (SPR)	[215]
Chronoamperometry	Carbon black NPs-SPE	KH_2_PO_4_	10^−8^	0.1	Drinking, river, aquarium, and waste water samples with satisfactory recovery values, absence of silicate interference, stable sensor (>3 months in a dry condition at RT), for online in-situ analysis.	[216]
I−V measurement	Silver/Graphene Composite/FET*	PO_4_^3–^	5–6000	0.2	Long-term stability, excellent reproducibility, and good selectivity, low-cost and applicable in real water samples	[217]
Amperometry	Paper CB-SPE*	K_2_HPO_4_	10–300	4	High reproducibility, long storage stability, reagentless, RSD < 6%	[218]
I−V measurement	Graphene/ionophore hybrid membrane ISFET*	PO_4_^3–^	-	2800	Good performance and selectivity, response time 10 s	[219]
Potentiometry	CuPc* Acrylate-Polymer/Silicon	K_2_HPO_4_	0.001–10	0.001	High specificity	[220]
Impedimetry	CuPc/Au electrode	Na_2_HPO_4_	10^−4^–1000	0.00838	-	[221]
Potentiometry	Zn^2+^/BPMP- Cu^2+^/BPMP*	K_2_HPO_4_	3–50	1 0.5	Good selectivity and stability	[222]
Amperometry	Platinum/Au nanowires Arrays	Thiamine pyrophosphate (TPP)	248–1456	45	Good selectivity, storage in citrate buffer at 4 °C in refrigerator and measurement every three days showed good stability	[223]
Voltammetry	Silver Nanowires/SPE	Na_2_HPO_4_	5–1000	3	Good repeatability and recovery	[224]
Potentiometry	Cobalt NPs-RGO*/GCE*	KH_2_PO_4_	1–10,000	-	Measurement in tap and well water samples	[225]
Voltammetry	Graphite SPE	KH_2_PO_4_	0.003–0.115	0.02	Dissolved phosphorus sensing in canal water samples	[226]
Potentiometry	ZnO NRs* FET	PO_4_^3–^	0.1–7000	0.05	-	[227]
Amperometry	PyOx*/Au nanowires	KH_2_PO_4_	12.5–1000	0.1	Good selectivity, stability > two weeks of repeated use in water samples (recovery of 96.67 ± 4.9%)	[228]

* PVC: Polyvinyl chloride, PANI: Polyaniline, CB-SPE: Carbon black screen printed electrodes, FET: Field-effect transistor, ISFET: Ion Sensitive Field Effect Transistor, CuPc: Copper Phthalocyanine, BPMP: 2,6-Bis{bis(2-pyridylmethyl)aminomethyl}-4-methylphenol, NPs: Nanoparticles, RGO: reduced graphene oxide, GCE: Glassy carbon electrode, NRs: Nanorods, PyOx: pyruvate oxidase.

**Table 7:** Electrochemical sensor for the detection of disinfectant in water.

Method	Electrode Modification	Analyte	LOD (µM)	Linearity (µM)	Ref
CV	CILE */CTS */Hb */GR-CuS * composite	TCA *	200	3000–64 × 10^3^	[243]
CV	CILE/CTS/Hb/3d GR *	TCA	130	400–26 × 10^3^	[233]
CV	CPE */CdO	TCA	2.3	3–230	[244]
SWV	GCE */Iron pthalocyanine/ZIF-8 *	TCA	1.89 × 10^−3^		[245]
EIS	GCE/MIP *	NDMA *	0.011	0.13–3.1	[246]
CV	CILE/Nafion/Hb/borondoped graphene qunatumdots	TCA, NaNO_2,_ H_2_O_2_	53	100–300 × 10^3^	[247]
CV	CILE/Nafion/Hb/ZnO-CNF *	TCA and NaNo_2_	1.33 × 10^3^	4 × 10^3^–150 × 10^3^	[248]
CV	CILE/Nafion/Hb/Co_3_O_4_-CNF	TCA, KBrO_3_ and NaNo_2_	1.33 × 10^3^	40 × 10^3^–260 × 10^3^	[249]
SWV	GCE/AgNp-Malic acid	TCA	30 × 10^−3^	01-2 & 4–100	[250]

* CILE: Carbon ionic liquid electrode, CTS: Chitosan, GR-CuS: graphene-Copper sulfide, 3d GR:3d Graphene, GCE: Glassy carbon electrode, MIP: Molecular imprinted polymer, ZIF-8: Zinc based metal-organic framework, Hb: Haemoglobin, CNF: carbon nanofibers, TCA: Trichloroacetic acid, NDMA: N-Nitrosodimethylamine.

**Table 8:** Overview of electrochemical sensors for some emergent contaminants.

Detection Method	Eltrode/Materials	Analyte	LOD (µM)	Dynamic Range (µM)	Comments	Reference
Amperometry	GCE-MWCNTs	Gallic acid	0.19	0.66–52.8	Cognac and brandie, sample dilution not required	[257]
SWV	SPE-CB *	Catechol, gallic acid, caffeic acid, and tyrosol	0.1, 1, 0.8, and 2	1–50 1–50 10–100 10–100	Foods and beverages	[258]
DPV	Press-produced CB transducer	Tyrosol Hydroxytrosol	20 6	10–75 10–75	Olive oil	[259]
DPV	GCE/nano-carbons-AgNPs	Gallic acid	0.063	0.5–8.5	Wine, sample dilution not required	[260]
DPV	GCE-GR/boron doped	Gallic acid	-	-	Tea infusion	[261]
DPV	GCE-GR reduced-Fe_2_O_3_/Chitosan	Gallic acid	0.51	1–0.01	Red/white, wine, sample dilution not required	[255]
Chronoamperometry	SPE-GR/PEDOT/PSS *	Trolox	0.59	5–30	Herbs and herbal beverages	[254]
DPV	Al_2_O_3_/AC-CPE *	Phenol	0.151	10–1000	Natural waters and olive oil	[262]
Amperometry	Nanoporous gold/Si wafer	Catechol	0.5	20–200	PBS	[263]
CV	Platinum–polytyramine composite/graphite substrate	Phenol	-	3 × 10^−2^–10 × 10^−3^	Industrial waste waters	[264]
SWV	Tyr *-AuNPs */SPE	Phenol	1.47	1.47–441	Sensitivity of 15.7 mA ppm^−1^ Regional water samples	[265]
Amperometry	Tyr-ZnO nanorods/Au	Phenol	0.6	0.6–2020–50	Sensitivity of 103.08 µA/mM	[266]
Stripping voltammetry	Nafion-Modified GCE	Phenol	0.001	0.008–10	Water samples	[267]
DPV	HEX-AET *-gold nanoparticles-Glassy Carbon Elecrode	Phenolic estrogens DES DE >BPA > HEX	0.0054 0.0033 0.0043 0.0054	-	Satisfactory linear range and selectivity	[268]
DPV	DNA aptamers/AuSPE *	17 β-estradiol	5 × 10^−7^ in tap water/7 × 10^−7^ in milk	1.5 × 10^−6^–10^−4^/10^−4^–0.07	Excellent selectivity	[269]
LSV	MIP */AuNPs/Au	17 β-estradiol	1.09 × 10^−9^	3.6 × 10^−9^–3.6 × 10^−3^	Broad linear range, high sensitivity, selectivity, and reproducibility, simple to fabricate, easy to operate.	[270]
DPV	MWCNTs/THI/AuNPs/SPWE	17 β-estradiol	0.0002	1.79 × 10^−7^–0.0018	Cost effective, wireless connection with smart phone	[271]
SWCASV	Polished silver solid amalgam electrode	Pyrethroid insecticide (beta-cyfluthrin (βCF))	-	1.2 × 10^−6^–3.0 × 10^−5^	Low detection limit with a high level of precision and accuracy	[272]
AdSDPV	SrTiO_3_/N-GNS *	Pharmaceutical compound: Diphenhydramine	0.0021	0.038–100.0 ×10^−6^	Good recoveries in synthetic pharmaceutical samples and human body fluids, good candidate for real application	[273]
Voltammetry	c-MWCNTs/GCE	BPA	5.0 × 10^−3^	(10–104) × 10^−3^	Recovery: 98.4–102.8%.	[274]
LSV	BSA/Anti-BPA/AuNP/MWCNT/GCE	BPA	8.7 × 10^−3^	0.01–1	Food fresh-keeping film. Recovery: 97.3–103%	[275]
Amperometry	BCNP/Tyr/Nafion/GCE	BPA	3.18 × 10^−3^	0.02–10	Water samples. Recovery: 96.67–108%.	[276]
DPV	SWCNTs/Poly-IL/GCE	BPA	10^−3^	5.0 × 10^−3^–30	Leaching from plastic drinking bottle.	[277]
DPV	Lac/Ag–ZnO/MWCNTs/CSPE	BPA	6 × 10^−3^	0.5–2.99	High level of precision and accuracy. BPA in plastic bottles.	[278]
Amperometry	Tyr-DAPPT–rGO/GCE	BPA	3.5 × 10^−3^	1.0 × 10^−3^–38	Commercial plastic drinking bottles.	[279]
DPV	MIPPy/GQDs/GCE	BPA	0.04	0.1–50	Tap and sea water samples, with recoveries of 94.5% and 93.7%	[280]
SWV	ZnO/CNT/IL/CPE	BPA	9 × 10^−3^	0.002–700	Food samples.	[281]
Derivative Voltammetry	MIP/CS/Gr/ABPE	BPA	6 × 10^−3^	8.0–2.0	Plastic bottled drinking water and canned beverages.	[282]
DPV	AuNP/Gr/GCE	BPA	5 × 10^−3^	0.01–10	Milk samples with recoveries of 105%.	[283]

* AuNPs: Gold nanoparticles, AuSPE: Gold screen printed electrode, CPE: Carbon paste electrode, MIP: Molecular imprinted polymer, CSPE: Carbon screen printed electrode, N-GNS: nano-cylindrical strontium titanate N-doped graphene, PEDOT:PSS: Poly(3,4-ethylenedioxythiophene):Polystyrene sulfonate, Al_2_O_3_/AC: aluminum oxide supported onto activated carbon, CB: Carbon Black, Tyr-ZnO nanorod: Tyrosinase immobilization on Zinc oxide nanorods, HEX: hexestrol, AET: 2-aminoethanethiol hydrochloride

**Table 9:** Overview of electrochemical sensors for dihydroxybenzene isomers.

Detection Method	Materials	LOD (µM) HQ, CC and RC	Dynamic Range (µM)	Comments	References
CV	CTAB-GO/MWCNTs/GCE	0.03 0.01 0.2	0.1–200 0.1–400 1–100	Tap water	[297]
DPV	WBC*/Au-850–15/GCE	0.002 0.004 -	0.008–7.0 0.01–7.0 -	Tap	[298]
DPV	MWCNTs@RGONR*/GCE	3.89 1.73 5.77	15–921 15–1101 15–1301	Tap, River	[299]
DPV	CN-F*/GCE	0.5 0.8 0.4	10–120 10–120 10–120	River	[300]
DPV	3D* CNT-Gr/AuNPs/GCE	0.8 0.95 0.1	0.0–80 0.0–80 0.0–80	Tap, River	[301]
DPV	Cu-MOF*-Gr/GCE	0.59 0.33 -	1.0–100 1.0–100 -	Tap water	[302]
DPV	UiO-66/MPC*-3/GCE	0.056 0.072 3.51	0.5–100 0.4–100 30–400	Tap-Lake	[303]
DPV	CNNS*-CNT/GCE	0.13 0.09 -	1–200 1–250 -	Tap	[304]
DPV	Chit*/MWCNTs/Ti_2_/GCE	0.06 0.07 0.52	0.4–276 0.4–159 3.0–657	River, Tap	[305]

* 3D: Three-dimensional, AuNPs: Gold nanoparticle, Chit: Chitosan, CNNS: Graphitic carbon nitride nanosheets, MOF: Metal-organic framework, MPC: Mesoporous carbon, CN-F: Carbon nano-fragment, RGONR: Reduced graphene oxide nanoribbon, WBC: White myoga ginger-derived biochar.
